
JOURNAL INFORMATION
==============================
NLM Title Abbreviation: J Child Orthop
Journal ID: J Child Orthop
NLM Journal ID: 101313582

Journal of Children's Orthopaedics

ISSN: 1863-2521
EISSN: 1863-2548
Publisher: SAGE Publications

ARTICLE INFORMATION
==============================
PMCID: PMC5145821
PMID: 27743244
DOI: 10.1007/s11832-016-0771-3
Article ID: 771
Article version: 1
Subjects: Abstracts

30th Annual Meeting of the Children’s Orthopaedics, Munich, 10–12 March 2016

Electronic publication date: 2016 Oct 14
Print publication date: 2016 Dec
Volume: 10
Issue: 6
First page: 717
Last page: 743
Copyright: © The Author(s) 2016
License: This article is distributed under the terms of the Creative Commons Attribution 4.0 International License (http:// creativecommons.org/licenses/by/4.0/), which permits unrestricted use, distribution, and reproduction in any medium, provided you give appropriate credit to the original author(s) and the source, provide a link to the Creative Commons license, and indicate if changes were made.
License URL: https://creativecommons.org/licenses/by/4.0/

issue-copyright-statement: © The Author(s) 2016

==============================

### Contents

Child Hip Disease: ePoster ... 718
Early Results After Arthroscopically-Assisted Repair of the TFCC in Children and Adolescents ... 718
Child Hip Disease: Lecture ... 718
Advanced Containment Procedures in Perthes Disease: Does Triple Pelvic Osteotomy Provide Best Coverage? ... 718
Gait Characteristics in Transverse Plane in Case of Offset-Loss After SCFE ... 719
Hip Development in Patients with Cerebral Palsy and Hip Dysplasia After Open Release of M. iliopsoas ... 719
In situ Fixation of Slipped Capital Femoral Epiphysis: Femoral Neck Growth is Not Impaired by Screw Fixation ... 720
Musculoskeletal Manifestations in Mucopolysaccharidosis Type I (Hurler Syndrome) Following Hematopoietic Stem Cell Transplantation ... 720
Not Fully Centered Femoral Head After Closed Reduction in Children with Developmental Dysplasia of the Hip: Immediate Re-Reduction is Not Necessary ... 720
Open Reduction of Hip Dislocation in Children with Arthrogryposis Multiplex Congenita: Mid Term Results ... 721
Outcome of Subtrochanteric Rotational Osteotomy for the Treatment of Reduced Femoral Antetorsion at End-of-Growth .. 721
Transinguinal Ultrasound Versus MRI for the Determination of Hip Position After Application of a Spica Cast ... 722
Valgus-, extension- and (de)rotation-Osteotomy of the Proximal Femur in Children with Spondyloepiphyseal Dysplasia Congenita ... 722
Norm Results and Norm Variants: Lecture ... 723
Femoral Torsion in Children with Proximal Focal Femur Deficiency and Arthrogryposis Multiplex Congenita ... 723
Surgical Hip Dislocation in Children and Adolescents: Clinical and Radiographic Results at 3 years Follow-UP ... 723
Knee Joint and Leg Axes: Lecture ... 724
3-D Gait Analysis After Limb Lengthening Using Motorized Intramedullary Nails ... 724
Dynamic Knee Joint Loading in Children and Adolescents with Idiopathic Genua Valga: Results after Surgical Correction of Axial Malalignment ... 724
*Efficacy of Percutaneous Epiphyseodesis Type Canale* ... 724
Guided Growth of Distal Medial Epiphysis of Tibia Using a Screw ... 725
Interobserver Variability in the Evaluation of X-ray of the Hand to Determine the Skeletal Age in Patients with PFFD and Fibula Hemimelia ... 725
MPFL Reconstruction Combined with a Modified Grammont Technique can Avoid Recurrent Patella Dislocation in Adolescent Patients with Patella-Femoral Dysplasia: a Case Series ... 726
Reduction of Adult Height: Hormonal Treatment or Epiphysiodeses ... 726
TAR Syndrom and Knee Contraction 90 Degrees Treated with TL-HEX ... 726
Technical and Surgical Orthopaedics Working Hand in Hand in Caring for Patients Suffering from Embryonic Dysplasia of the Knee and Malposition of the Axis as in the Gollop–Wolfgang Complex ... 727
Temporary Epiphysiodesis for Correction of Leg Length Discrepancy: Comparison of RigidTacks™, Eight-Plates™ and Blount Staples ... 727
Torsional osteotomy of Tibia: Review of 391 Cases ... 728
Two Novel Knee Size-Adjusted Parameters for Patella Maltracking in Children ... 728
Knee Joint and Leg Axes: How to Treat: Case Description ... 729
Preliminary Results of Anatomic All-Inside All-Epiphyseal Single Bundle Anterior Cruciate Ligament Reconstruction in Tanner Stage 1 and 2 Patients with Open Physes ... 729
Neuroorthopedics: ePoster ... 729
Maximum Deformity of Hip and Knee Joint in a 15 Year-Old Refugee Child from Eritrea with Cerebral Palsy ... 729
Neuroorthopedics: Lecture ... 729
Development of a Classification System for Wheelchair Maintenance and Seat Positioning for Patients with Neuro-Muscular Diseases and Dysmorphia of Skeleton ... 729
Gastrocnemius Function in Crouch Gait: a Direct Muscle Imaging Study in Children with Bilateral Spastic Cerebral Palsy ... 730
Hereditary Sensory Autonomic Neuropathy Type IV: An Attempt to Elaborate a Therapeutic Concept Based on Literature and Clinical Experiences ... 730
Hip Surveillance in Cerebral Palsy: Lessons Learned? ... 731
Long-Term Results of Single-Event Multilevel Surgery in Children with Spastic Hemiplegia ... 731
Long-Term Therapy with Intrathecal Baclofen Improves Life Quality in Children with Severe Spastic Cerebral Palsy ... 731
Possibilities and Limitations of the Newest Generation of Video Based 3D Gait Analysis Systems ... 732
Predictors for Pelvic Anterior Tilt after Patella Tendon Shortening in Children with Cerebral Palsy .......... 732
The Elbow in Arthrogryposis Multiplex Congenita .......... 732
The Results of Preoperative Halo-Gravity Traction for the Treatment of Severe Spinal Deformity in Children .......... 733
Foot Deformities: ePoster .......... 733
Results After Percutaneous Muscle Release of a Flexible Plat Foot in a 8 year old Child with Cerebral Palsy .......... 733
Wait and Watch vs. Early Operative Therapy in an Extremely Low Birth Weight Newborn with Necrotic Feet and Fingers .......... 734
Foot deformities: Lecture .......... 734
Changes in Foot Motion and Plantar Pressure After Tibialis Anterior Tendon Transfer for Clubfoot Recurrence .......... 734
Early Complications After Subtalar Arthroereisis in the Treatment of the Juvenile Flatfoot .......... 734
*Early Treatment of Juvenile Hallux Valgus* .......... 735
Serial Ankle Casts for Patients with Idiopathic Toe Walking: Effects on Functional Gait Parameters .......... 735
Surgical Treatment of Talocalcaneal Coalition in Childhood and Adolescence: Experience and Unfavourable Results in 80 Cases During a 13-Year-Period .......... 735
Treatment of Ponseti-Clubfoot-Relaps with an Abduction-Night-cast Versus TAT .......... 736
Paediatric Orthopaedic Pitfalls: ePoster .......... 736
Aneurysmal Bone Cyst of the Patella .......... 736
Osteopetrosis: Case-Report of Patient with Genua Valga et Recurvata and Subtrocanteric Femur Fracture, Treatment Options and Their Complications .......... 737
Pronation Deformity of Forearm Following Fracture both Bones of Forearm: Relavence of Pronator Teres in the Management of Fracture .......... 737
Paediatric Orthopaedic Pitfalls: Lecture .......... 737
A Minimum of 2 year Follow up of 22 EOS Patients Who Were Treated With 2nd Generation MCGR .......... 737
Analysis of Complications and Pitfalls of the LCP Pediatric Hip System .......... 738
Arthrodiatasis with Hinged Monoplanar External Fixator for Perthes Disease: A Technical Tip. Unpopular does not Mean Ineffective .......... 738
Body Height Prediction and Detection of Growth Patterns after Systemic Chemotherapy in Children with Osteo- and Ewing's Sarcoma .......... 739
Diastrophic Dysplasia: a Long Term Follow-up of Orthopedic Pediatric Treatment in 26 Cases .......... 739
Paediatric Orthopaedic Evaluation of Children Suffering from Nephropathic Cystinosis at the Interdisciplinary Cystinosis Clinic in Traunstein .......... 739
Surface Optometry in Scoliosis Comparison of 102 Scoliosis Patients and a Peer Group of 100 .......... 740
Ultrasound Diagnosis of Fractures: State of the Art .......... 740
How-to-treat Cases: Lecture .......... 741
Neglected Hip Fracture after Percutaneous Fixation of Epiphysiolysis Cap. Fem. in a 10 years Old Girl .......... 741
Nora's Lesion on the Tibia of a 4 Years Old Boy .......... 741
Osteofibrous Dysplasia Campanacci of the Ulna .......... 741
How-to-Treat Cases: How to Treat: Case Description .......... 742
Case Report: Infant from Libya with Amputation of the Lower Leg and Pseudarthrose of the Femoral Bone after Gunshot Wound .......... 742
Complex Reconstruction of the Hip Joint after Purulent Coxitis with Coincidence of DDH with Coxa Magna in the Contralateral Hip .......... 742
Supramalleolar Valgus Deformity with Partial Necrosis of the Distal Tibial Epiphysis .......... 743

### Child Hip Disease: ePoster

***Child hip disease: ePoster VKO-110***

#### *Early Results After Arthroscopically-Assisted Repair of the TFCC in Children and Adolescents*

S. Farr[1], U. Zechmann[2], R. Ganger[1], W. Girsch[2]

[1]Orthopädisches Spital Speising, Abteilung für Kinderorthopädie und Fußchirurgie, Wien, Österreich, [2]Orthopädisches Spital Speising, Wien, Österreich

***Question*** The purpose of this study was to report our preliminary results after arthroscopically-assisted repair of peripheral triangular fibrocartilage complex (TFCC) tears in adolescent patients.
***Methodology*** All children and adolescents who underwent arthroscopically-assisted repair of a Palmer 1B tear were identified and prospectively evaluated after a mean follow-up of 1.3 years. The postoperative assessment included documentation of clinical parameters, pain score (visual analogue scale, VAS), grip strength and completion of validated outcome scores (Modified Mayo Wrist Score, MMWS; Disabilities of the Arm, Shoulder and Hand Inventory, DASH). A total of 12 patients (four males, eight females) with a mean age of 16.3 years at the time of surgery were evaluated.
***Result*** The mean VAS decreased significantly from 7.0 to 1.7 after the procedure. We observed a significant increase of the MMWS after surgery; however, MMWS was still significantly lower at final follow-up when compared to the contralateral side. A mean postoperative DASH score of 16 indicated an excellent outcome after the procedure. DASH Sports and Work Modules showed fair and good overall outcomes in the short-term, respectively. Grip strength averaged 86 % of the contralateral side at final follow-up, with no significant difference being found between both sides.
***Conclusion*** Arthroscopically-assisted repair of peripheral TFCC tears in adolescents provided predictable pain relief and markedly improved functional outcome scores. Concomitant pathologies may have to be addressed at the same time to eventually achieve a satisfactory outcome. Sports participation, however, may be compromised in the short-term and should therefore be resumed 6 months postoperatively.

### Child Hip Disease: Lecture

***Child hip disease: Lecture VKO-198***

#### *Advanced Containment Procedures in Perthes Disease: Does Triple Pelvic Osteotomy Provide Best Coverage?*

R. Pospischill, C. Weiss, R. Ganger

Orthopädisches Spital Wien Speising, Abt. für Kinderorthopädie und Fusschirurgie, Wien, Österreich

***Question*** Indication for surgical treatment methods in older patients with severe LCPD is still in discussion. Proximal femoral varus osteotomy (VO) or the Salter osteotomy (SO) may provide adequate containment in moderate LCPD but can be problematic in older patients where advanced procedures such as the triple pelvic osteotomy (TPO) are considered as the method of choice. The aim of this study was to evaluate postoperative coverage parameters after primary hip containment surgery in patients older than 6 years with severe LCPD.

***Methodology*** A retrospective analysis was performed of all patients hospitalized at our department between January 2002 and October 2014 who underwent surgery due to the diagnosis LCPD. We identified all patients with (1) VO, SO + VO or TPO (2) age >6 years at time of surgery (3) no underlying neurogenic disorder or previous surgery. 65 patients were left for statistical analysis. The CE-angle, the acetabular index and the extrusion index were assessed using pre- and postoperative standing AP radiographs of the pelvis.

***Result*** For the cohort the mean age at the time of surgery was 7.74 years. 70 % were classified Herring B/C or C. In the VO group no significant changes of the coverage parameters were found. TPO and SO + VO showed statistically highly significant improvement of all postoperative measurements ($p < 0.001$).

When comparing the parameters between the treatment groups, the postoperative mean CE angle was higher in the TPO group and showed an increase of 16.7° compared to 11.1° in the SO + VO group. In the TPO group the postoperative EI decreased by 13.06 % compared to 10.9 % in the SO + VO group. The CE angle and AI corrected significantly better in the TPO than in the VO group ($p = 0.049$, $p = 0.008$). At skeletal maturity we could not identify any significant difference concerning preoperative and final measurements between the three procedures.

***Conclusion*** Our results confirm significantly better femoral coverage immediately after surgery using TPO compared to VO, and our data suggest TPO tending to cover the femoral head better than SO + VO. However, the lack of statistically significant superiority of TPO at skeletal maturity could not only be attributed to the natural history of severe Perthes cases. Investigations of a larger cohort of patients at skeletal maturity in each treatment group is necessary to confirm the present findings.

***Child hip disease: Lecture VKO-141***

### *Gait Characteristics in Transverse Plane in Case of Offset-Loss After SCFE*

B. Westhoff, S. Hummel, D. Rosenthal, C. Zilkens, R. Krauspe

University of Duesseldorf, Medical Faculty Department of Orthopaedics, Duesseldorf, Deutschland

***Question*** Residual deformity of the femoral head after SCFE might be accompanied by a loss of femoral offset and may lead to femoroacetabular impingement (FAI). Especially during hip flexion the offset-loss might be relevant. It is hypothesized that during phases of the gait-cycle, when the hip is in flexion, the offset-loss is compensated by an increased external rotation.

***Methodology*** 36 patients with the diagnosis of SCFE (23 male, 13 female, mean age 23.2 years, mean BMI 27.7), who were treated by pinning-in situ, were included. Inclusion criteria: (1) uni- or bilateral SCFE, (2) age >18 years, (3) BMI <40, (4) follow-up > 3 years. Exclusion criteria: any other disorder associated with gait deviations. Femoral offset was measured on axial radiographs by the alpha-angle. Patients were divided into 3 offset-groups (OG): OG1 ($n = 11$)—alpha angle <55°, OG2 ($n = 13$)—alpha angle 55–75°, OG3 ($n = 12$)—alpha angle >75°. 3D-gait-analysis was performed with a VICON 512 system. Spatiotemporal, kinematic and kinetic parameters were evaluated and compared to an control group with no gait deviations ($n = 40$, 7 male, 33 female, mean age 28.4 years, mean BMI 21.9). For statistics Pearson correlation analysis and a one-way ANOVA of the controls and offset groups with Dunnett-T3 post hoc comparisons were performed.

***Result*** In comparison to the controls patients walked with increased external rotation of the hip, especially at the point of maximum hip flexion during terminal swing phase (90–100 % of the gait cycle): The mean external hip rotation during this period differs significantly across the groups (controls: 6.19° (5.29), OG1: 17.30° (6.31), OG2: 19.25° (7.95), OG3: 20.28° (8.74), $p \leq .001$). External hip rotation was ascending with increasing offset-loss.

At initial contact (0 % of the gait cycle) patients showed an increased external rotation of the hip as well: all OGs differ significantly from the controls [controls: 6.77° (5.44), OG1 16.81° (8.50), OG2 19.19° (8.53), OG3 20.47° (9.48), $p \leq .016$] and external hip rotation increases with offset-loss.

During hip extension (40–60 % GC) patients walked in external hip rotation [OG1 4.52° (7.91), OG2 8.48° (8.72), OG3 11.01° (8.84)] whereas controls showed an internal hip rotation [−2.50° (5.81)]. OG2 and OG3 differ significantly from the controls ($p = .004$; $p = .001$).

***Conclusion*** Residual deformity of the proximal femur after SCFE leads to increased external hip rotation during gait, with decreasing femoral offset more external rotation is needed to avoid FAI.

***Child hip disease: Lecture VKO-113***

### *Hip Development in Patients with Cerebral Palsy and Hip Dysplasia After Open Release of M. iliopsoas*

V. Gattung, N. Herzig, M. Hauptmann, F. Oczipka, V. Frimberger, J. Schmid, M. Poschmann, P. Bernius

Schön Klinik München Harlaching, Zentrum für Kinder- und Neuroorthopedics, München, Deutschland

***Question*** Beside M. adductor longus and knee bending muscles, M. iliopsoas is one of the main muscles leading to hip displacement in hip dysplasia in patients with cerebral palsy.

As previously presented, our data in children with cerebral palsy aged from 1 to 6 years examined from 2011 to 2014 showed an improvement or at least a stabilization of hip situation after percutaneous release of hip surrounding muscles.

We now show the results in patients who needed an open release of M. iliopsoas in addition to percutaneous release.

***Methodology*** We retrospectively examined 27 hips in 14 patients suffering from cerebral palsy aged from 3 to 18 years (mean 8.3) operated from 2011 to 2014 with a preoperative RMI of at least 30 %. The severity of cerebral palsy was classified according to gross motor function classification system (GMFCS). Patient's GMFCS ranged from 3 to 5. RMI was measured in X-rays pre- and postoperatively in a follow up of 2–45 month (mean 18.1 months). Patients all had a release of M. iliopsoas.

We compared these patients with our patient group who had a percutaneous muscle release (results presented in VKO congress 2015 in Frankfurt).

***Result*** Overall, we saw a decrease of RMI from 50.5 to 46 %. Depending on mobility scale, we saw a decrease from 40.7 to 38 % in patients with GMFCS 2–3 ($n = 6$), from 49 to 42 % in patients with GMFCS 4 ($n = 6$) and a decrease from 58.5 to 54 % in patients with GMFCS 5 ($n = 10$).

Depending on severity of preoperative hip displacement, we found a decrease of RMI from 30 to 23 % ($n = 7$) when initial RMI was lower than 39 %, a decrease from 41 to 40 % ($n = 7$) when RMI was between 40 and 49 % and a decrease from 66.9 to 61 % ($n = 13$) when RMI was higher than 50 %.

Compared to our group who underwent percutaneous myofasciotomy without release of M. iliopsoas, we found a significant decrease of RMI in patients with GMFCS 4. There was a significant and highly significant decrease of RMI when initial RMI was between 40 and 49 % or higher than 50 %.

Patients who needed a release of M. iliopsoas had a higher initial RMI and were older compared to patients who only needed a percutaneous release.
***Conclusion*** In contrast to our data shown for patients after percutaneous muscle release, patients who needed a release of M. iliopsoas with initial RMI over 40 % had no benefit and further hip reconstruction will be needed. We conclude that percutaneous muscle release should be performed in an early stage to improve hip situation, as long as there is no contraction of M. iliopsoas.

***References***
[1] Cornell et al., (1995), The hip in cerebral palsy
[2] Flynn et al., (2002), Management of hip disorders in patients with cerebral palsy

***Child hip disease: Lecture VKO-178***

### *In situ Fixation of Slipped Capital Femoral Epiphysis: Femoral Neck Growth is Not Impaired by Screw Fixation*

S. Urlaub, R. Taurman, H. Reichel, J. Wölfle-Roos

Orthopädische Universitätsklinik am RKU Ulm, Orthopädie, Ulm, Deutschland

***Question*** Ever since screw fixation was first used in patients with slipped capital femoral epiphysis (SCFE), there has been concern if longitudinal growth of the femoral neck might thus be impaired. In a matched-pair study no clinically relevant difference of femoral neck growth between k-wire and screw fixation was seen when comparing the asymptomatic contralateral hip treated prophylactically in patients with unilateral SCFE. However, it is unclear if these results can be applied to the primarily affected hip in SCFE. Thus, the aim of this matched-pair analysis was the comparison of femoral neck growth of the primarily affected hip in SCFE between k-wire and screw fixation.
***Methodology*** All 15 patients (female:male = 2:13) who had undergone screw fixation of SCFE between 2002 and 2013 were matched according to age, gender, slipping angle and time to follow-up to another 15 patients with k-wire fixation. The length of the femoral neck of the symptomatic hip was measured in parallel to either screw or k-wire from the apex of the femoral head to the opposite cortical bone. The ratio of the femoral neck length measured directly after surgery and on follow-up was defined as femoral neck growth.
***Result*** There was no significant difference between groups with regard to age ($13.5 \pm 1.2$ vs. $13.3 \pm 1.4$ years, $p = 0.50$), slipping angle ($19.2 \pm 8.2°$ vs $19.93 \pm 6.8°$, $p = 0.71$) and follow-up ($1.6 \pm 0.6$ vs. $1.6 \pm 0.7$ years, $p = 0.92$). We found a significantly higher femoral neck growth in patients with screw fixation ($4.69 \pm 2.8$ %) when compared to k-wire fixation ($2.8 \pm 3.0$ %; $p = 0.025$ matched Wilcoxon Test).
***Conclusion*** In this matched-pair study the assumption that longitudinal growth of the femoral neck is impaired by screw fixation was not confirmed. We even found a slightly but significantly higher longitudinal growth of the femoral neck in screw fixation when compared to k-wire fixation. Thus, screw fixation remains a viable option for in situ fixation of SCFE.

***Child hip disease: Lecture VKO-144***

### *Musculoskeletal Manifestations in Mucopolysaccharidosis Type I (Hurler Syndrome) Following Hematopoietic Stem Cell Transplantation*

S. Breyer[1], M. Schmidt[2], U. Löbel[3], I. Müller[4], K. Ullrich[2], R. Stücker[1], S. Yarar[5], N. Muschol[2]

[1]Altonaer Children's Hospital, Pediatric orthopedics, Hamburg, Deutschland, [2]UKE, Pädiatrie, Hamburg, Deutschland, [3]UKE, Department of Diagnostic and Interventional Neuroradiology, Hamburg, Deutschland, [4]UKE, Clinic of Pediatric Hematology and Oncology, Division for Stem Cell Transplantation, Hamburg, Deutschland, [5]UKE, Department of Trauma, Hand and Reconstructive Surgery, Hamburg, Deutschland

***Question*** Hematopoietic stem cell transplantation (HSCT) is the treatment of choice in young Hurler patients. Despite the preservation of neurocognitive function and improved life expectancy, the beneficial effect on the skeletal system is limited. Orthopedic complications are one of the most disabling factors following HSCT aggravating the need for new treatment strategies. The study summarizes musculoskeletal manifestations in 19 transplanted Hurler patients.
***Methodology*** Data were obtained retrospectively. Patients' charts for physical examinations of the joint range of motion (JROM) of shoulders, elbows, hips and knees were reviewed. Radiographic evaluations of thorax, spine, pelvis and hands were performed. MRI scans of the craniocervical junction (CCJ) were analyzed to determine odontoid hypoplasia and the prevalence of craniocervical stenosis (CCS).
***Result*** Nineteen Hurler patients (10 females, 9 males) with an average age of 8.1 years (range 2.5–23.8) at the latest follow-up, who underwent allogenic HSCT between 1991 and 2012, were assessed after an average follow-up period of 6.4 years (range 0.7–22.5). Seventeen patients achieved long-term engraftment with a full donor chimerism (10 patients) or mixed chimerism between 16.7 and 59.3 % (7 patients). Two patients developed graft failure 1 and 10 years after HSCT, respectively. The majority of patients showed a steady state resp. improvements in the JROM of knees (31 % resp. 63 %), hips (47 % resp. 40 %) and elbows (56 % resp. 38 %) leading to a full range of motion in 44–69 % of joints. In patients with graft failure progressive restrictions in JROM were noted. Assessments of CCJ by MRI showed stable or improved diameters in 67 % of patients. Correction or stabilization of odontoid hypoplasia was found in 64 % during follow-up. However thoracolumbar kyphosis, hip dysplasia as well as genua valga were progressive despite HSCT.
***Conclusion*** Joint mobility, odontoid hypoplasia and craniocervical stenosis might stabilize or improve in Hurler patients following HSCT. Nevertheless, despite beneficial effects on some musculoskeletal manifestations, skeletal complications are frequently seen. Frequent multi-disciplinary follow-ups in specialized centers are essential. Novel therapeutic approaches (e.g. anti-inflammatory drug) are required to improve the musculoskeletal outcome.

***Child hip disease: Lecture VKO-146***

### *Not Fully Centered Femoral Head After Closed Reduction in Children with Developmental Dysplasia of the Hip: Immediate Re-Reduction is Not Necessary*

H. Huber[1,2], S. Tharakan[3], S. Dierauer[4], C. Kellenberger[5], L.E. Ramseier[2]

[1]Kantonsspital Winterthur, Kinderorthopädie, Klinik für Orthopädie und Traumatologie, Winterthur, Schweiz, [2]Kinderpital Zürich, Kinderorthopädie, Klinik für Kinderchirurgie, Zürich, Schweiz, [3]Kinderspital Zürich, Klinik für Kinderchirurgie, Zürich, Schweiz, [4]Kinderspital Zürich, Kinderorthopädie, Klinik für Kinderchirurgie, Zürich, Schweiz, [5]Kinderpital Zürich, Abteilung für Bilddiagnostik, Zürich, Schweiz

***Question*** Closed reduction and spica cast application under general anaesthesia is a standard procedure in the treatment of DDH. MRI is the gold standard to confirm correct reduction. However, some hips appear to be in a slightly decentred position and the question arises, if a immediate re-reduction is necessary.
The goal of our study is to establish a measurement method for hip centring in the axial plane and to evaluate the natural course of initial mild subluxation by means of repeat MRI examination 2 weeks after spica cast application. In addition, we wanted to evaluate if hip abduction in the cast has an influence on the evolution.
***Methodology*** Between 2008 and 2014, a total of 43 patients underwent closed reduction and spica cast application due to DDH or congenital/teratolgic hip dislocation. 24 of these patients showed good centring on the MRI, 7 patients had an immediate cast change. 12 patients showed a slightly decentred position in the axial plane and MRI was repeated 2 weeks later. These cases were reviewed retrospectively. We propose as a quantitative measurement method of hip centring the hip centring index (HCI), the ratio between femoral head radius (r) and distance between the centre of the femoral head and the central point in the depth of the acetabulum (a).

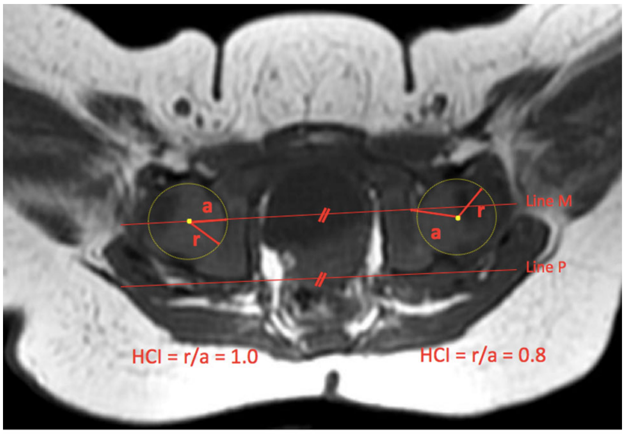

Line P: line tangentially to both ischiums
Line M: parallel to line P through the centre of the centred hip (right hip)
r: Radius of the femoral head
a: Distance between femoral head centre and crossing point between line M and acetabulum

The value of the HCI (r/a) would be 1 if the femoral head is in contact with the depth of the centre of the acetabulum. Hip abduction was measured also on axial MRI sequences.
***Result*** HCI on the initial MRI was 0.74 (range 0.56–0.93) and improved to 0.86 (range 0.67–1) after 2 weeks, whereas no significant change could be observed on the healthy side [mean HCI 0.87 (range 0.76–1) vs. 0.89 (range 0.83–1) at FU]. The mean abduction of the dysplastic hips in the cast as seen on MRI was 43° (range 12°–55°). Improvement of HCI ($p = 0.004$) was statistically significant correlated with the amount of abduction. In 2 of 12 hips the spica cast was changed due to surgeon’s decision. These two hips showed a HCI below 0.7 and a hip abduction smaller than 45°.
***Conclusion*** Slight decentring of the hip after spica cast application improves after 2 weeks in most cases. An insufficient hip abduction in the cast might be a reason for a more generous cast change in the case of hip subluxation.

***Child hip disease: Lecture VKO-158***

### *Open Reduction of Hip Dislocation in Children with Arthrogryposis Multiplex Congenita: Mid Term Results*

S. Marx, J. Wolz, T. Kastner, S. Nader

Schön Klinik, Kinderorthopädie, Vogtareuth, Deutschland

***Question*** Arthrogryposis multiplex congenita is a rare disease with multiple joint contractures and often occurs in combination with other syndromal disorders.
One of the common findings in AMC aside stiffness of the joints is the congenital dislocation of the hip.
In the last decades several authors encouraged hip surgery by presenting quite good results.
This report aims to analyze mid term results after surgical management of uni- and bilateral dislocation of the hip in children with AMC.
***Methodology*** Between 2006 and 2014 we performed open reduction, varus osteotomy and derotation of the femur, Pemberton osteotomy and capsulorhaphy in 17 children with 18 hips (16 children unilateral, 1 child bilateral). One patient got lost to follow up.
Preoperatively 5 patients presented with hip luxation Tönnis III° and 12 patients presented with hip luxation Tönnis IV.
***Result*** The period of follow-up ranged from 2 months to 9 years with a mean of 4.7 years. At the last follow up the average acetabular index was 20° and the average centre-edge angle was 20°. There was an increase of stiffness or decrease of motion in 3 patients, all children could walk with the same support as preoperatively. The clinical results were graded according to the McKay’s classification, showing 9 good 5 fair and 3 poor results.
There was no major complication especially no need for re-reduction in our cases.
***Conclusion*** As shown by other authors, we experienced good results with open reduction in children with AMC with uni- or bilateral hip dislocation. Except for the challenging surgery itself because of the pathological variances, we experienced no complication after surgery. However, the mid-term results revealed avascular necrosis in 23 % of our cases. In our experience even older children benefit from open reduction. Long term follow-ups are needed to prove the exact benefits. The chosen scoring system does not take in account the congenital disability of the child with arthrogryposis.

***Child hip disease: Lecture VKO-120***

### *Outcome of Subtrochanteric Rotational Osteotomy for the Treatment of Reduced Femoral Antetorsion at End-of-Growth*

D. Burger[1], L.E. Ramseier[2], S. Dierauer[3], H. Huber[4]

[1]Kantonsspital Winterthur, Winterthur, Schweiz, [2]Kinderspital Zürich, Kinderorthopädie, Klinik für Kinderchirurgie, Zürich, Schweiz, [3]Kinderpital Zürich, Kinderorthopädie, Klinik für Kinderchirurgie, Zürich, Schweiz, [4]Kantonsspital Winterthur, Kinderorthopädie, Klinik für Orthopädie und Traumatologie, Winterthur, Schweiz

***Question*** Reduced femoral antetorsion may cause out-toeing gait and also femoro-acetabular impingement. Femoral rotational osteotomy is a popular intervention to correct this deformity in children and adolescents. On short term, this improves internal rotation of the hip, but no mid- or long-term follow-up exists. We were interested if a significant loss of correction would occur during the last years of remaining growth and if factors exist that would predict a major loss of correction (age at surgery, body weight).
***Methodology*** We could collect data from 16 patients (27 hips) treated with subtrochanteric rotational osteotomy between 2004 and 2009 (mean age at surgery 13.4 years, range 8.6 to 16.6 years). Clinical measurement of hip internal rotation before surgery, 3 months postoperatively and at latest follow-up at skeletal maturity were reviewed. Body weight was available for all of these patients at the latest follow-up.

***Result*** Three month after subtrochanteric osteotomy, internal rotation of the hip improved from a mean preooperative value of 10.0° (range 0°–20°) to 38.0° (range 30°–60°). At skeletal maturity mean hip internal rotation decreased again [mean 22.8°, range 10°–40° ($p = 0.001$)]. Both boys and girls lost equally in internal rotation (15.2°). Considering the age of the patients, boys operated before age 14 lost significantly more internal rotation (mean loss 22°) compared to boys older than 14 years at date of surgery (mean loss 9°, $p = 0.008$). Girls younger than 12 also showed an increased loss of internal rotation compared to girls older than 12 years at date of the surgery [mean loss 21° vs 11° ($p = 0.188$)]. Patients with Body-mass index (BMI) >20 kg/m$^2$ showed a comparable loss of internal rotation compared to children with a BMI <20 kg/m$^2$ [mean loss 14.9 vs 14.7° ($p > 0.05$)].

***Conclusion*** After correction of reduced femoral antetorsion by means of femoral rotational osteotomy, recurrent loss of internal rotation occurs during the last years of growth. Especially girls operated before age 12 and boys before age 14 are prone to loose correction. Although we cannot differentiate if loss of internal rotation is due to loss of femoral antetorsion or due to local changings at the femoral head neck-junction or acetabular rim, this loss should be taken into account for the timing of surgery.

***Child hip disease: Lecture VKO-200***

### *Transinguinal Ultrasound Versus MRI for the Determination of Hip Position After Application of a Spica Cast*

O. Eberhardt[1], T. von Kalle[2], F. Fernandez Fernandez[1], C. Peterlein[3], T. Wirth[1]

[1]Klinikum Stuttgart Olgahospital, Orthopädische Klinik, Stuttgart, Deutschland, [2]Klinikum Stuttgart Olgahospital, Radiologisches Institut, Stuttgart, Deutschland, [3]Universiätsklinikum Marburg, Zentrum für Orthopädie und Unfallchirurgie, Marburg, Deutschland

***Question*** MRI is the most common method to evaluate the hip position after closed or open reduction in DDH treatment. In this study we evaluated the efficacy of transinguinal ultrasound versus MRI to determine the femoral head position after open or arthroscopic reduction and application of a spica cast.

***Methodology*** 6 patients with 9 dislocated hips were treated by hip arthroscopy or open reduction. There were 5 girls and 1 boy with 9 dislocated hips after failed closed reduction. 7 hips were reduced by hip arthroscopy and 2 hips by open reduction. To determine the femoral head position after reduction and application of a spica cast transinguinal ultrasound and MRI were performed and evaluated by our radiology department. The 16 ultrasound investigations were compared to the 16 hips evaluated by the MRI's. Two children had bilateral dislocations so that we could also compare dislocated hips on ultrasound images and MRI's. The images were blinded reviewed by three of the authors (a radiologist, a senior orthopaedic surgeon and an orthopaedic resident). Anatomical landmarks were identified on both images (femoral head, pubic bone, femoral neck). The grade of dislocation was determined by the classification according to Szuzuki.

***Result*** The interpretation of the ultrasound images and the MRI images by the three study participants were the same in 15 hips and were the same of the findings by the radiologist department immediately after the surgical procedures. 16 images were correctly assessed by the radiologist. 15 images were correctly assessed by the senior orthopaedic surgeon and the orthopaedic resident. Only one ultrasound image was misinterpreted by the orthopaedic surgeons. The important anatomical landmarks could be identified in all 16 hips. The Szuzuki classification was incorrect assessed in one case.

***Conclusion*** This study has shown that transinguinal ultrasound is a save method to determine the hip position after hip reduction. Even in difficult cases after open reduction or arthroscopic reduction with sophisticated anatomical preconditions the efficacy of transinguinal ultrasound is comparable to MRI.

***Child hip disease: Lecture VKO-116***

### *Valgus-, extension- and (de)rotation-Osteotomy of the Proximal Femur in Children with Spondyloepiphyseal Dysplasia Congenita*

M. Kröner, S. Nader

Schön Klinik Vogtareuth, Klinik für Kinderorthopädie, Vogtareuth, Deutschland

***Question*** A common problem in children with congenital spondyloepiphyseal dysplasia is the multidirectional malposition of the proximal femur. The malposition is split in (1) the coxa vara, (2) the pathological torsion of the femur and (3) a deficit in the extension of the hip. The children suffer from limited walking distance caused by a rapid fatigue and pain. In the examination a hyperlordosis of the lumbar spine is seen, an in-toeing or out-toeing gait and the Trendelenburg-sign. Hence, there is a high risk of falling with all possibilities of a severe trauma.

***Methodology*** The aim of the study was to compare the results of the multidirectional correction osteotomy of the proximal femur fixed by a LCP Paediatric Hip Plate (Fa. Synthes) in patients with spondyloepiphyseal dysplasia. All patients with spondyloepiphyseal dysplasia who got a valgus-, extension- and (de)rotation-osteotomy of the proximal femur in our hospital were reexamined. We reviewed retrospectively the radiographic and clinical data.

***Result*** In 48 months (02/2010–02/2014) we operated on 9 patients 16 hips. The average age was 10.3 years. The Neck-shaft angle, the Hilgenreiner epiphyseal/Sharp angle, the sacrofemoral angle and the ROM have been evaluated preoperatively and postoperatively. The age at date of surgery was documented as well as the date of removal of the plate. Furthermore other operations in history were noted just as complications and/or re-operations.

The mean Neck-shaft angle has been increased to 46.3° and the mean sacrofemoral angle has been reduced to 29°. The surgical treatment lead to an increased extension but to no decrease in the ROM. The rate of complications was low.

***Conclusion*** Children with spondyloepiphyseal dysplasia congenita benefit a lot from a valgus-, extension- and (de)rotation-osteotomy of the proximal femur. In these rare cases this type of surgical treatment is a good possibility to improve the hip conditions. The patients show a great benefit by an increased walking distance, a better standing position and a reduced Trendelenburg-sign.

## Norm Results and Norm Variants: Lecture

***Norm results and norm variants: Lecture VKO-123***

### *Femoral Torsion in Children with Proximal Focal Femur Deficiency and Arthrogryposis Multiplex Congenita*

M. Klein, H. Forkl, S. Nader

Schön Klinik Vogtareuth, Kinderorthopädie, Vogtareuth, Deutschland

***Question*** PFFD is a rare congenital disorder of the femur often associated with a leg length inequality, malalignment of the axis and abnormity of the femoral torsion. A PFFD often goes with a fibular hemimelia, instability of the knee joint and deformities of the feet. AMC is a complex of symptoms characterized by multiple congenital joint contractures. In 80 % of the cases the hips are affected. Hip pathologies are dislocations, subluxations and contractures. Due to this deformities affected children are dependent on surgeries and/or orthopedic aids such as orthosis. This study provides information about the ratios of the torsion of the lower extremities in children with PFFD and AMC.
***Methodology*** Between March 2011 and April 2015 18 children with PFFD and 12 with AMC underwent a measurement of the torsion with MRI or CT scan. The torsion of the acetabulum and femur, the rotation of the knee and the instability index (Mc Kibbin Index) were evaluated. The 30 children, 18 girls and 12 boys, had a mean age of 11 (3–26) years. 14 children with PFFD and all children with AMC were dependent on orthopedic aids.
***Result*** In the children with PFFD the mean anteversion of the acetabulum (AV) of the affected leg was 12° (opposite side 17°), the mean antetorsion (AT) of the femoral neck was 7° (opposite side 24°) and the mean external torsion of the lower leg was 23° (opposite side 28°). In the AMC group the mean AV was 17° for both sides, the mean AT was 10° (left) and 0° (right) and the mean external torsion of the lower leg was 38° (left) and 36° (right). The Mc Kibbin Index of the affected side of the children with PFFD was 19 (opposite side 41). In the children with AMC the Mc Kibbin Index was 27 (left) and 17 (right).
***Conclusion*** Children with PFFD do have a reduced antetorsion of the femoral neck on the affected leg compared to the other side. Children with AMC do have a reduced AT on both sides. According to our experience a derotation osteotomy simplifies the fitting of orthetic devices. A very high or low Mc Kibbin Index is associated with more pain and a higher rate of osteoarthritis. In our children with PFFD the instability index is strikingly lower on the affected side compared to the other leg. In children with AMC the index is reduced on both sides. These results keep us running a torsion measurement for a correct planning of a derotation osteotomy to simplify the providing of an orthosis and to improve the quality of life of children with PFFD and AMC by reducing pain and osteoarthritis.

***Norm results and norm variants: Lecture VKO-177***

### *Surgical Hip Dislocation in Children and Adolescents: Clinical and Radiographic Results at 3 years Follow-UP*

N. Guindani[1], O. Eberhardt[1], T. Wirth[1], M.F. Surace[2], F.F. Fernandez[1]

[1]Olgahospital - Klinikum Stuttgart, Orthopädische Klinik, Stuttgart, Deutschland, [2]Dipartimento Di Biotecnologie e Scienze Della Vita, University Of Insubria, Clinica Ortopedica e Traumatologica, Varese, Italien

***Question*** Hip deformities due to paediatric pathologies and complex abnormalities leading to femoroacetabular impingement (FAI), functional limitation and eventually premature osteoarthritis (OA) may be approached with surgical hip dislocation (SHD) [1]. The aim of this study is to evaluate the clinical, radiographic short-term results and complications after SHD in young patients (≤18 years).
***Methodology*** Clinical and radiographic outcomes were assessed in patients who underwent a SHD Ganz-type approach between 2008 and 2012. Diagnosis included Perthes (LCPD), epiphysiolysis (SCFE), FAI, osteonecrosis (ON), Ombredanne disease (MHE), pigmented villonodular synovitis (PVNS). Preoperative and postoperative clinical data, radiographs, modified Harris hip score (mHHS), non-arthritic hip score (NAHS) and short form 12 (SF-12) questionnaires were evaluated.
***Result*** After a mean 3 years follow-up (range 0.5–6 years) were evaluated 53 hips (51 patients): 15 LCPD, 13 ECF, 18 FAI, 4 MHE, 3 miscellaneous. Mean age at surgery was 14 years (range 10–18 years). Through this approach femoral head-neck osteoplasty (33), Dunn-type osteotomy (10), labrum refixation (5), synoviectomy (2), femoral head mosaicplasty (2) open reduction and fixation for SCFE (1) were performed, eventually in association with pelvic (3), intertrochanteric (4) osteotomy or flake repair (2). At FU, better outcome scores were obtained and 90 % of the patients were satisfied with the procedure. ON progression was observed in four cases and in three further patients a THA was implanted; another with LCPD and preoperative osteonecrosis had a functional anchilosis.

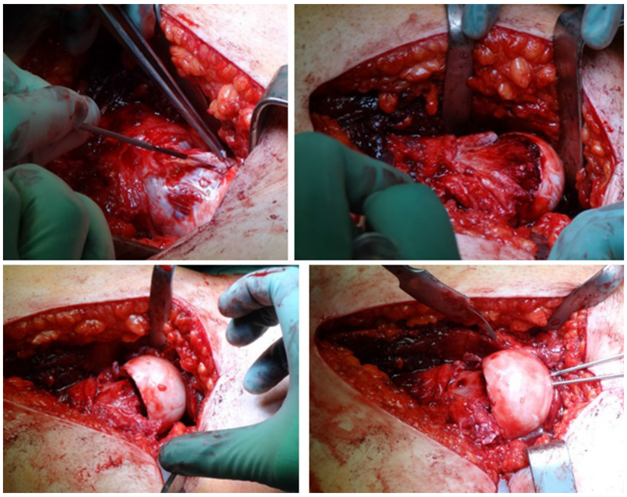

***Conclusion*** Severe hip deformities in paediatric/young patients may be approached with SHD. After 3 years FU results are comparable to previous studies [2–4] and patients have a high rate of satisfaction, however the effectiveness of those procedures have to be proved on the long term. Results and complications seem to be related with preoperative lesion(s) and type of treatment.

***References***
[1] Rebello G, Spencer S, Millis MB, Kim YJ., (2009), Surgical Dislocation in the Management of Pediatric and Adolescent Hip Deformity., Clin Orthop Relat Res. 2009 Mar;467(3):724–31.
[2] Ziebarth K, Zilkens C, Spencer S, Leunig M, Ganz R, Kim YJ., (2009), Capital Realignment for Moderate and Severe SCFE Using a Modified Dunn Procedure., Clin Orthop Relat Res (2009) 467:704–716
[3] Tibor LM, Sink EL., (2013), Risks and Benefits of the Modified Dunn Approach for Treatment of Moderate or Severe Slipped Capital Femoral Epiphysis., J Pediatr Orthop 2013;33:S99–S102
[4] Albers CE, Steppacher SD, Ganz R, Siebenrock KA, Tannast M., (2012), Joint-preserving Surgery Improves Pain, Range of Motion, and Abductor Strength After Legg-Calvé-Perthes Disease, Clin Orthop Relat Res (2012) 470:2450–2461

### Knee Joint and Leg Axes: Lecture

***Knee joint and leg axes: Lecture VKO-190***

#### *3-D Gait Analysis After Limb Lengthening Using Motorized Intramedullary Nails*

K. Burger[1], A. Handlbauer[1], A. Kranzl[2], R. Ganger[1], C. Radler[1]

[1]Orthopaedic Hospital Speising, Vienna, Department of Pediatric Orthopaedics, Wien, Österreich, [2]Orthopaedic Hospital Speising, Vienna, Laboratory for Gait and Movement Analysis, Wien, Österreich

***Question*** Several studies showed that limb length discrepancy (LLD) leads to compensatory mechanisms in gait. Leg lengthening with the aim of improving symmetry in gait using motorized intramedullary devices has gained popularity in recent years. The aim of this study was to evaluate the gait pattern of patients who underwent limb lengthening with a motorized intramedullary nail.
***Methodology*** Between 2007 and 2014 a total of 50 patients with the mean age of 21 years (18–52) were treated with a motorized intramedullary lengthening device (Fitbone®). At the end of treatment 30 patients (16 male, 14 female, mean age: 20 years) were evaluated with a 3-D gait analysis. Preoperative limb length discrepancy ranged from 21 to 55 mm (median 30 mm). Ten patients underwent tibia lengthening (group A) and 20 patients femoral lengthening (group B). A discrepancy of limb length of more than 5 mm remained in 8 patients (range −2 to 12 mm). We analysed gait parameters, kinematic and kinetic data. A reference group ($n = 26$) was used as a control group. Differences between lengthened and contralateral leg were investigated to evaluate improvement of symmetry using *t* test for sample pairs. The data of group of patients and control group were verified for homogenity and compared with unpaired t-test.
***Result*** The comparison of the lengthened to unaffected leg showed significant less maximum ankle performance and maximal dorsiflexion in swing phase in group A. This was also found in group B. Further, in group B the position of pelvis at the affected leg at initial contact was lower and more external rotation in femur was seen. Comparison of the lengthened leg to reference group showed less ankle movement and dorsiflexion in swing phase and increased valgus deformity of the affected leg in group A. In group B lower position of pelvic in frontal plane and similar alterations as in group A were seen in the lengthened leg.
***Conclusion*** Typical compensatory mechanisms and gait patterns described in literature for patients with LLD were no longer found in our analysis. However, deficits in ankle joint movement, maximum dorsiflexion and slight pelvic obliquity in frontal plane persisted despite leg length equalisation without significant sagittal alterations in hip, knee or ankle. The results underline the importance of physiotherapy and gait training after equalization of leg length.

***Knee joint and leg axes: Lecture VKO-109***

#### *Dynamic Knee Joint Loading in Children and Adolescents with Idiopathic Genua Valga: Results after Surgical Correction of Axial Malalignment*

S. Farr[1], B. Attwenger[2], J. Hahne[2], R. Ganger[1], A. Kranzl[2]

[1]Orthopädisches Spital Speising, Abteilung für Kinderorthopädie und Fußchirurgie, Wien, Österreich, [2]Orthopädisches Spital Speising, Wien, Österreich

***Question*** Previous studies have shown that children and adolescents with genua valga exhibit considerable compensatory mechanisms during gait cycle. These children demonstrated significantly decreased internal knee valgus moments and different transverse plane gait patterns such as decreased external knee rotation and increased external hip rotation. The purpose of this study was to re-evaluate these children after surgical correction of their lower limb alignment using hemiepiphysiodesis in order to determine if knee joint loading and rotational gait patterns were normalized after the intervention.
***Methodology*** We evaluated 20 children (8 female, 12 male), with a mean age of 13.6 years (range 11.8–14.8 years) at the time of explantation of the hemiepiphysiodesis device ("8-plate") and gait analysis. Nineteen bilateral, and one unilateral case were included in the analysis, accounting for a total of 39 limbs. Radiographic indices according to Paley et al. (e.g. MAD, LDFA, MPTA) and predefined gait parameters (frontal knee moments, foot progression angle, hip and knee rotation, knee valgus angle) were documented, and pre-postoperative comparisons were performed.
***Result*** Surgical correction of limb alignment resulted in a significant normalization of frontal knee moments and frontal knee angle ($p < 0.0001$). Foot progression angle and hip rotation, however, showed no significant changes ($p = 0.804$, $p = 0.966$). Moreover, knee rotation changed significantly towards an increased internal rotation ($p = 0.008$). All radiographic parameters were significantly improved ($p < 0.001$). Compared to a reference cohort of healthy children, there eventually remained a significant decreased external knee rotation ($p = 0.006$) as well as a significantly increased hip external rotation ($p > 0.0001$).
***Conclusion*** Our results suggest that correction of limb axis in children with idiopathic genua valga is highly beneficial to improve and normalize knee joint loading during gait analysis. Distinct rotational gait patterns have not been influenced by these surgical procedures and may reflect pre-existing gait abnormalities in this patient cohort. Results indicate that the decreased external knee rotation might be compensated by a muscle-driven increase of external hip rotation.

***Knee joint and leg axes: Lecture VKO-192***

#### Efficacy of Percutaneous Epiphyseodesis Type Canale

H. Grünewald, D. Dovi-Akue, K. Kuminack, A. El Tayeh

Universität Freiburg, Klinik für Orthopädie und Unfallchirurgie, Sektion Kinderorthopädie, Freiburg, Deutschland

***Question*** The percutaneous epiphyseodesis type Canale (ED) is an established methode to treat leg length discrepancy. The literature describe it as a save and reliable procedure. Our clinical experience doesn't show regularly a leg length compensation after the ED. Aim of the study was to measure the leg length discrepancy after ED in the knee and with it the efficacy of the ED.
***Methodology*** A retrospective studie of cases was undertaken from 04/2002 to 04/2014. All the patients got a ED in the knee (distal femur, proximal Tibia and proximal Fibula) because of their leg length discrepancy. The residual growth and with it the time of operation was determined by the atlas of Greulich and Pyle and by Multiplier manner of Paley. We required a follow-up at least 1 year postoperative. The limb leg discrepancy was postoperative measured by standing blocks, we renounce to do an X-ray. The limb length discrepancy preoperatively was determined by full length standing AP radiograph. The statistical comparison was done with SPSS through descriptive statistics.
***Result*** We identified a total of 29 cases. Sex ratio was approximately equal (M:F 15:14). The preoperatively measured average leg length discrepancy was 2.5 cm. Postoperatively (averaged 2.5 years) we measured in average 1.3 cm leg length discrepancy. Consequently, the ED achieved a leg length compensation about 45 %.

***Conclusion*** 2.5 years after Epiphyseodesis type Canale 45 % of the initial leg length discrepancy is compensated. The reason of the not sufficient correction of the leg length is not clarified enough. In future there have to be more studies to determine the reasons or to develop a save procedure to compensate the leg length discrepancy. The limits on our study was at the one hand the short follow-up, and on the other hand, the missing X-ray to determine the exact leg length discrepancy. We renounce it conscious because of radiation protection reasons.

In conclusion, the percutaneous epiphyseodesis type Canale is not a save and reliable procedure to compensate leg length discrepancy.

***Knee joint and leg axes: Lecture VKO-171***

### *Guided Growth of Distal Medial Epiphysis of Tibia Using a Screw*

C.U. Dussa, D. Lewens, V. Maestri, L. Döderlein

Orthopädische Kinderklinik, Kinderorthopaedie, Aschau i. Chiemgau, Deutschland

***Question*** The aim of the study was to review the effectiveness, rate of correction, rebound effect, complications of correction of valgus deformity of distal Tibia using a screw.

***Methodology*** All the patients who had explantation of screw in the distal medial tibia between 2008 and 2014 were included in the study. Patient demographics, diagnosis, implantation and explantation dates were recorded. A standardised radiograph of the distal tibia was used to measure the distal lateral angle of tibia (lDTA) as recommended by Paley D. An lDTA <86° was considered pathological. The pre-implantation and pre-explantation lDTA were measured on the radiographs of each limb. The rate of correction, complications and recurrence was recorded in every case. All the patients were followed for a minimum of 12 months or until end of growth to exclude growth plate damage.

***Result*** In all 146 Patients were operated in the period between 2008 and 2014. In all cases a 4.5 mm stainless steel or titanium screw was sued for Epiphyseodesis of distal medial tibia. Of these patients, the explantation was done in 71 Limbs (51 Patients). Of these 20 cases were bilateral. The diagnosis was very diverse and could be grouped into 9 major groups. The mean age of patients was 10.1 years. There were 38 male and 14 female patients. The mean pre-implantation lDTA was 79.98°. The mean pre-explantation lDTA was 87.81°. The mean correction was 7.83°. The mean correction period was 14.87 months, with a mean correction rate of 0.52°/month. The fastest correction rate was achieved in the Clubfoot group with 0.66°/month and the slowest in the bone dysplasia group with 0.32°/month.

Complications included overcorrection in 2 cases, complicated explantation needing osteotomy of medial malleolus. Failure to achieve full correction was seen in 4 cases. Residual deformity was seen in three patients but was expected.

***Conclusion*** Irrespective of the aetiology, the Epiphyseodesis with a screw is effective to correct the valgus deformity in the region of distal tibia. This was observed in our study group which was very heterogenous. The technique of Epiphyseodesis is easy and can be safely carried out with a screw without major problems. A permanent Epiphyseodesis as a result of a bone bridge was not seen in any of our patients. The correction rate is predictable irrespective of the aetiology at about 0.5°/month. In our study the maximum valgus deformity that was corrected was 17°. Explantation is quick and easy but is not free of difficulties.

***Knee joint and leg axes: Lecture VKO-129***

### *Interobserver Variability in the Evaluation of X-ray of the Hand to Determine the Skeletal Age in Patients with PFFD and Fibula Hemimelia*

H. Forkl, S. Marx, K. Koch, S. Nader

Schön Klinik Vogtareuth, Klinik für Kinderorthopädie, Vogtareuth, Deutschland

***Question*** In patients with PFFD or Fibula Hemimelia treatment is focused on equalizing leg length difference. To obtain an exact prognosis of expected body height and leg length in maturity the determination of the skeletal age is essential. This study points out the interobserver variability in evaluating the bone age by X-ray of the hand and the consequences in prediction of the remaining growth.

***Methodology*** In 73 patients with PFFD and/or Fibula Hemimelia 129 a.p. X-rays of the hand were taken (9/2011–11/2015) to determine the bone age. The mean chronological age was 7 years (±3.3) at the date of X-ray. The X-rays were evaluated by 3 different independent doctors using the atlas of Greulich and Pyle. The existing leg difference was measured from a.p. X-ray of both legs.

The estimated limb discrepancy and body height in maturity were calculated by using the multiplier method. The results were registered statistically.

***Result*** Observer 1 detected a mean skeletal age of 6.6 (±3.3) years, observer 2 and 3 of 6.5 (±3.3; ±3.4) years. The interrater reliability showed an intra-class correlation coefficient of 0.996. This indicates a good accordance of the observers. The skeletal age was found significantly ($p < 0.005$) lower than the chronological age by each observer.

Calculated for the chronological age the mean estimated height was 172 cm (±10.6), the mean estimated length difference of the femur was 6.9 and 4.8 cm of the tibia. In each observer a significant difference was shown regarding the estimated body height and femoral length difference by using the evaluated skeletal age instead of the chronological age for the prognosis in maturity. The interrater reliability was high ($r > 0.9$).

Increasing age of the patients showed an approximation of the skeletal age and chronological age in each observer. In 40 % of the patients a dissociated development of the hand was found.

***Conclusion*** In this study a high interobserver accordance in the evaluation of the skeletal age using the atlas of Greulich and Pyle by the 3 observers was found. The detected skeletal age was significantly lower compared to the chronological age. This strongly influences the predicted height and length discrepancies.

Frequently dissociated skeletal development of the hand was found. Especially in younger children the difference between the skeletal and the chronological age was higher, often associated with a dissociated skeletal hand. In these cases the evaluation of the X-ray by an experienced team is essential.

***Knee joint and leg axes: Lecture VKO-152***

### *MPFL Reconstruction Combined with a Modified Grammont Technique can Avoid Recurrent Patella Dislocation in Adolescent Patients with Patella-Femoral Dysplasia: a Case Series*

M. Sperl[1], H. Wegmann[2], K. Regvar[1], R. Eberl[2], M. Svehlik[1], C. Würnschimmel[1], T. Kraus[1]

[1]Medizinische Universität Graz, Klinische Abteilung für Kinderorthopädie, Univ. Klinik für Kinder- und Jugendchirurgie, Graz, Österreich, [2]Medizinische Universität Graz, Univ. Klinik für Kinder- und Jugendchirurgie, Graz, Österreich

***Background*** Patella dislocation is the most common cause of knee joint hemarthrosis during growth. Risk factors such as patella femoral dysplasia and malalignment are identified for recurrent dislocation. In the growing skeleton, treatment options are limited due to open physis.
***Purpose*** This case series presents the long term outcome of MPFL reconstruction in combination with a modified Grammont Technique.
***Methodology*** Between 2009 and 2013, 7 knees in 6 patients (4 male, 2 female) were treated by MPFL reconstruction in combination with a distal soft tissue realignment. Before and at mean 45 (20–61) months after surgery, patients were evaluated by clinical examination, functional scores (Tegner, Kujala), and radiographs of the knees.
***Result*** Mean age at time of operation was 16 years (14–17). Five knees show a trochlear Dysplasia Dejour B, one a type A and one type C. Mean of TTTG was 18.5 mm (16–21.9 mm). Kujala score improved significantly from preoperatively 52.17 (STD 28.25) to 92.67 (STD 9.99) at follow-up ($p < 0.04$). Tegner Score improved from 2.5 preoperatively (STD 2.74) to 6 (STD 1.79) at follow up ($p < 0.06$). There was no re-dislocation.
**Discussion** In a growing skeleton, patella dislocation and recurrent instability is attributed to predisposing factors such as bone dysplasia and malalignment. The MPFL is the main constraint against lateralisation of the patella. Treatment options are, among others, MPFL reconstruction and distal realignment procedures.
***Conclusion*** MPFL reconstruction combined with a modified Grammont procedure seems to prevent patella instability in immature patients with patellofemoral dysplasia and malalignment.

***Knee joint and leg axes: Lecture VKO-112***

### *Reduction of Adult Height: Hormonal Treatment or Epiphysiodeses*

R. Roedl[1], B. Vogt[1], G. Gosheger[2], H. Tretow[1], F. Schiedel[1]

[1]Universitätsklinikum Münster, Kinderorthopädie, Deformitätenrekonstruktion und Fußchirurgie, Münster, Deutschland, [2]Universitätsklinikum Münster, Allgemeine Orthopädie und Tumororthopädie, Münster, Deutschland

***Question*** Hormonal treatment for extreme tall stature is well established since 1930. Oestrogen doses were reduced from 1000 to 100 µg per day. Hormonal treatment reduces mainly the growth of long bones.
2004 first publications (Lancet 2004; 364: 1513–18) report serious undesirable long term effects.
Fertility problems are reported and risks of malignant tumours are discussed.
2010 a publication (Int J Pediatr Endocrinol 2010:740629) was introducing epiphysiodesis as an alternative treatment.
However critical discussion of this alternative regarding its effect on body proportions, timing of epiphysiodeses and operative technique is needed.
***Methodology*** Based on the data of the Zurich longitudinal growth study the height ratio of sitting height to subischial leg length was evaluated.
The ratio of 20 extremely tall individuals (height >3SD) was calculated.
Timing of Epiphysiodeses to reduce leg length at least 10 cm was estimated based on the Anderson and Green Data.
Possible operative techniques—permanent and non permanent epiphysiodeses—and their specific complication profile are discussed.
***Result*** The calculating example for a tall girl (average values + 4SD) results in a sitting height of 98.7 cm and a subischial leg length of 93.1 cm.
This ends in a height ratio of 1.06 (normal Value 1.14 ± 0.05).
The height ratio of the measured tall patients was 9.89 (9.5–1.08). This suggests that tall people have relatively long legs.
A leg shortening of 10 cm in a girl with a height ratio of 1.06 and a total height of 191 cm will result in a height ratio of 1.19.
Remaining growth of the distal femur is 6.4 cm and of the proximal tibia 4.1 cm in a 9 year old girl. That means timing of epiphysiodeses needs to be relatively early.
Permanent epiphysiodeses is only one time surgery but burning bridges. Non permanent epiphysiodeses needs implant removal but allows to recalculate after 2 years. In any doubt the possibility of adjustments and reopening of the physes remaines.
***Conclusion*** Patients with extremely tall stature have longer legs than individuals with average stature according to the height ratio. Therefore reduction of adult height by epiphysiodeses is a serious treatment option for those patients.
Because of the increasing knowledge of side effects of hormonal treatment it should be offered to patients and their parents as an possible alternative.
Because of the need of an early decision making non permanent epiphysiodesis should be considered to keep the possibility to revise the decision.

***Knee joint and leg axes: Lecture VKO-162***

### *TAR Syndrom and Knee Contraction 90 Degrees Treated with TL-HEX*

A. Helmers, S. Götz, K. von dem Busche

EWK Spandau, Kinderorthopädie, Berlin, Deutschland

***Question*** Case report: 4 year old girl with severe knee contraction and clubfeet was be unable to walk. The patient was mobile sitting in a buggy with the 1 year old brother.
***Methodology*** The feet where treated with the Ponseti Method and a tenotomy of the achillo tendon. After treating the feet the girl had surgical osteotomies of both knees and treatment with a TL-HEX (hexapod ring fixated with extra small rings and ultrashort struts) to stretch the contraction of the knees 1 mm per day and to reconstruct the position of both epiphysis. After the treatment with the Ponseti-Method and Hexapod Fixateur the girl was supported with 2 orthosis to fix the leg and feet position.
***Result*** After the treatment with the Ponseti-Method, the Hexapod Fixateur and the support with 2 orthosis the girl was be able to walk and climb stairs. She had physiotherapy started to mobilize her with an anterior walker and later without any external support.
***Conclusion*** Patients with TAR Syndrom are be able to walk after stretching both knees, in this case using the TL-HEX with ultrashort struts and very small rings. With that system we where able to treat both legs simultaneously.

***Knee joint and leg axes: Lecture VKO-138***

### *Technical and Surgical Orthopaedics Working Hand in Hand in Caring for Patients Suffering from Embryonic Dysplasia of the Knee and Malposition of the Axis as in the Gollop–Wolfgang Complex*

J. Wolz[1], M. Reiter[2], S. Kappl[1], S. Nader[1]

[1]Schön Klinik Vogtareuth, Klinik für Kinderorthopädie, Vogtareuth, Deutschland, [2]Orthopädietechnik Chiemgau Vogtareuth, Vogtareuth, Deutschland

***Question*** Demands in orthopaedic protheses are growing with the age of children. Our case report shows that complex deformities can be dealt with by dynamic ortheses to maintain mobility.

***Methodology*** A 6 year old boy with tibial and patella aplasia, luxation of the fibula and a medial duplication of the femur. Normal motoric history in infancy, first femoral orthesis when 19 months old with an extension deficit of 80° and therefore able to walk. Development of pressure marks. In the age of three surgical removal of the femoral duplicate, neurolysis and adaption of the muscles. Second femoral orthesis. After some time development of a knee extension deficit of 90° leading to further problems. In the age of five dorsomedial release of the head of fibula, reducing the extension deficit to 10°. Moderate delayed wound healing, luxation of the fibula and a shortage in blood vessels and nerves. Hence no replacement of fibula possible. Therefore third femoral orthesis. Again pressure marks. In the age of six orthesis number four providing a balanced weight reducing pressure marks. The special construction of the orthesis maintains a more dynamic pace by retreating the knee joint. After 3 months fracture of the metal joints made of titanium and the shaft of the orthesis. Hence, change of material (stainless-steel).

***Result*** The patient is able to walk freely, stairs can be taken. The knee can be moved freely. The geometric order of pivots provide a maximum of steadiness. Change of position like sitting to walking is possible. This type of construction fits the function of an expensive knee prothesis. No pressure marks due to full weight bearing at the distal part of the femur. Knee extension increases stretching of the tissue, also in preparation for another surgery to increase the balance of weight. Higher stability due to the new material of stainless-steel and due to strengthening the shaft.

***Conclusion*** A dynamic femoral orthesis is also possible in patients with complex embryonic reductions of the lower extremities. Limits due to material can be resolved in development of new, more stabilizing basic material in combination with new techniques in fabrication. Further scientific research is compulsory to consolidate the methods.

***Knee joint and leg axes: Lecture VKO-156***

### *Temporary Epiphysiodesis for Correction of Leg Length Discrepancy: Comparison of RigidTacks$^{TM}$, Eight-Plates$^{TM}$ and Blount Staples*

B. Vogt[1], M.-T. Kleine-König[1], G. Gosheger[2], H. Tretow[1], M. Horter[1], F. Schiedel[1], R. Rödl[1]

[1]Universitätsklinikum Münster, Kinderorthopädie, Deformitätenrekonstruktion und Fußchirurgie, Münster, Deutschland, [2]Universitätsklinikum Münster, Allgemeine Orthopädie und Tumororthopädie, Münster, Deutschland

***Question*** Epiphysiodesis (ED) is an established surgical procedure for moderate leg length discrepancies (LLD) between 2 and 5 cm in growing children. Since decades both permanent percutaneous procedures and temporary Blount stapling have been widely used for this purpose. In recent years the range of indication of 2-hole-plates like the eight-Plate$^{TM}$ that were originally developed for angular deformity (AD) correction of was extended to LLD. There is lack of studies in literature analysing the usage of 2-hole-plates for LLD correction. The shift of the fulcrum for correction outside the growth plate—a decisive advantage in AD correction—leads to central deformations of the femoral and tibial joint line when using medial and lateral plates. However, iatrogenic coronal and sagittal AD resulting from an asymmetric growth arrest at the physis when correcting LLD are still severe complications of all mentioned procedures.

***Methodology*** In consequence of these problems our study group developed new devices for temporary ED that consist of anatomically preformed staples with cannulated legs ensuring the simple and precise implantation technique of the 2-hole-plates. For AD correction staples with a flexible crossbar (FlexTack$^{TM}$) are used that constitute the tension band effect at the edge of the growth plate by analogy with the 2-hole-plates. To provide a complete and evenly distributed arrest over the entire growth plate when correcting LLD and with that to avoid the mentioned complications concerning iatrogenic angular deformities and joint line changes, staples with a rigid crossbar and reinforced corners (RigidTack$^{TM}$) are implanted.

A comparison of a prospective cohort of XX pat. (m = XX, f = XX; age = ~XX.X y.) with XX consecutive RigidTacks$^{TM}$ and historical cohorts of XX pat. (m = XX, f = XX; age = ~XX.X y.) with XX eight-Plates$^{TM}$ and XX pat. (m = XX, f = XX; age = ~XX.X y.) with XX Blount staples (follow up period ~ X.X y. (max. X.X y.) was done.

***Result*** Based on comparable initial conditions all three methods reached significant LLD-reductions with similar correction potentials. However, the correction speed was faster using rigid implants, especially the RT. General complication rates were comparable, but implant associated problems were significantly lower using guided devices. There was a remarkable number of iatrogenic AD in the BS-(coronal plane, esp. varus) and EP-group (sagittal plane, recurvation), while no iatrogenic AD had to be observed using RT so far

| | RigidTack$^{TM}$-group | eight-Plate$^{TM}$-group | Blount-group |
|---|---|---|---|
| **LLD preoparative [cm]** | 2.4 ± 1.6 | 2.9 ± 1.2 | 2.7 ± 1.4 |
| **LLD postoperative [cm]** | 1.4 ± 0.6 | 1.1 ± 1.3 [-0.6; 7.5] | 1.2 ± 1.2 |
| **Correction speed per physis [mm / months / physis]** | 0.9 ± 0.6 | 0.5 ± 0.8 | 0.6 ± 0.7 |
| **Implant associated complications (loosening, migration, breakage)** | 1 (4.3 %) | 4 (10.5 %) | 20 (14.6 %) |
| **Wound infection / Joint effusion** | 1 (4.3 %) | 1 (2.6 %) | 7 (5.1 %) |
| **Neurovascular complications** | 0 (0 %) | 0 (0 %) | 1 (0.7 %) |
| **Iatrogenic angular deformity (varus, valgus, recurvation)** | 0 ( 0 %), 0 (0 %), 0 (0 %) | 3 (7.9 %), 3 (7.9 %), 15 / 31 (48.4 %) | 35 (25.6 %), 12 (8.8 %), 33 / 84 (39.3 %) |

***Conclusion*** The RigidTack™ is a reasonable synthesis of staples and cannulated screw/plate devices. It seams to be even more simply handled for implantation reducing time for surgery and radiation exposure.
Due to the biomechanical improvements described faster corrections and lower rates of implant associated problems could be achieved. No joint line changes and iatrogenic AD had to be observed so far. However, these are preliminary results with a short term follow up. Further investigations are needed to confirm these encouraging data.

***Knee joint and leg axes: Lecture VKO-140***

### *Torsional osteotomy of Tibia: Review of 391 Cases*

C.U. Dussa, J. Radu, F. Paulitsch, L. Döderlein

Orthopädische Kinderklinik, Kinderorthopaedie, Aschau i. Chiemgau, Deutschland

***Question*** Torsion of Tibia may be congenital or acquired as part of deformity of foot or thigh. The aim of this study is to review the incidence of torsional deformities of tibia in relation to specific disease condition, associated limb deformities, surgeries performed along the correction of tibia torsion, type of implant, complications and outcome.
***Methodology*** A retrospective study was performed on the torsional correction of Tibia between 2007 and 2014. The data was collected using the case notes and radiographs. The data collected were demographics of the patient, diagnosis, deformity (single level or multi-level), type of torsional correction, time of correction, type of implant, time of union, complications and outcome.
***Result*** From 2007 until 2014, 392 surgeries were performed in 212 Patients to correct the torsional deformities of tibia. One Patient was excluded to lack of data. Of these were 180 males and 131 females. In 61 % of the cases, the diagnosis was a neuro-orthopaedic condition in 36 % of the cases was a non neuro-orthopaedic condition. An associated foot deformity was present in 73 % of the cases and was corrected in 71 % of the cases. A rotation osteotomy was performed in 61 % of the cases. In rest of the cases a derotation osteotomy was performed. The most common type of fixation was a DC Plate in 67 %, where as in others K-wires or a locking plate were used. In all 17 (4 %) complications and 6 under corrections were seen.
***Conclusion*** Tibia torsion may exist independently or as part of multilevel deformity of the limb, which may either be concealed, and therefore overseen (e.g. foot deformity or in femoral torsions) or revealed and therefore obvious. A concealed deformity becomes revealed when the foot is corrected and therefore should be looked for. From our experience, no additional investigation is necessary for the diagnosis. Acute correction with conventional implants is safe. Routine implants such as DC-Plates or K-Wires give good results in over 95 % of the cases.

***Knee joint and leg axes: Lecture VKO-125***

### *Two Novel Knee Size-Adjusted Parameters for Patella Maltracking in Children*

F. Wagner[1], J. Matussek[1], G. Maderbacher[1], S. Winkler[1], S. Anders[1], J. Hubertus[2], J. Grifka[1], A. Keshmiri[1]

[1]Universität Regensburg, Orthopädische Klinik für die Universität Regensburg, Regensburg, Deutschland, [2]Ludwig-Maximilians-Universität, Klinikum der Universität, Klinik und Poliklinik für Kinderchirurgie, München, Deutschland

***Question*** Anterior knee pain and patellar dislocation due to patella maltracking is a common finding in children. So far, the exact influence of the individual trochlea geometry and rotational limb alignment on patellar kinematics in children is poorly understood. Additionally, most radiological parameters do not consider the knee size of the growing child. The aim of this study was to develop anatomical knee size adjusted parameters for patellar maltracking on the basis of magnetic resonance imaging (MRI).
***Methodology*** MRI's of knees from 23 children with patella instability (PI) and 25 children without any history of patella-associated disease was analysed. Conventional rotational limb alignment as well as different trochlea parameters and ratios in defined measurement planes were generated and calculated by two blinded and independent observers. Only MRI from patients with open epiphyseal plates were considered.
***Result*** Between both groups significant differences were found for tubersositas tibea—trochlear grove distance (TTTG), tuberositas tibiae—posterior cruciate ligament insertion distance (TTPCL), trochlea sulcus angle (TSL) and lateral trochlea angle (LTIA). As a new parameter tibial head diameter/TTPCL ratio (THD/TTPCL) differed significantly between groups (3.6 $\pm$ 0.14 SEM in the control group vs. 3.2 $\pm$ 0.01 SEM in the PI group; $p = 0.043$). As an additional parameter TTPCL/LTIA (lateral trochlea inclination angle) was significantly higher in the PI group (1.24 $\pm$ 0.05 SEM vs. 0.97 $\pm$ 0.05 SEM; $p = 0.001$).
***Conclusion*** THD/TTPCL represents a novel measure describing malpositioning of the patellar ligament insertion determined solely by bony tibial landmarks and independent of knee positioning during MRI examination. TTPCL/LTIA equally determines maltracking for the femur. We describe two novel parameters, that are knee size adjusted and therefore ideal age-independent measures in the growing skeletal system of children. Combining these parameters might give additional guidance in surgical treatment of patella maltracking in the future.

## Knee Joint and Leg Axes: How to Treat: Case Description

***Knee joint and leg axes: How to treat: Case description VKO-142***

### *Preliminary Results of Anatomic All-Inside All-Epiphyseal Single Bundle Anterior Cruciate Ligament Reconstruction in Tanner Stage 1 and 2 Patients with Open Physes*

H. Wegmann

Universitätsklinikum Graz, Abteilung für Kinder- und Jugendchirurgie, Graz, Österreich

***Background*** Anterior cruciate ligament (ACL) injuries are increasing in the juvenile population. Historically, ACL reconstruction in skeletally immature patients was not recommended because of potential iatrogenic physeal injury resulting in growth arrest, limb-length discrepancies and angular deformities. Physeal-sparing alternative techniques including inter-substance suture or extra-anatomical reconstruction have been developed but showed unfavorable long-term outcome. Recently, all-epiphyseal reconstruction has been introduced, restoring the intra-articular anatomy while minimizing the risk of physeal injury.

***Purpose*** Presentation of preliminary results after anatomic all-inside all-epiphyseal single bundle anterior cruciate reconstruction in 11 patients.

***Patients and Methods*** Between 2013 and 2015, 11 prepubescent patients with Tanner stage 1 and 2 underwent ACL reconstruction using soft tissue grafts. Preoperatively the International Knee Documentation (IKDC) score was evaluated and anterior drawer sign objectified using a rolimeter. A single bundle all-inside all-epiphyseal technique using Arthrex retrodrills© was applied. Grafts were fixed with Arthrex TightRope RT©. Assessment of outcome included rolimeter, IKDC score, clinical and radiological examination.

***Results*** Mean age at surgery was 11.8 years (10–15 years). The following autologous hamstring grafts were used: n = 7 quadruple semitendinosus tendon, n = 1 quadruple semitendinosus tendon augmented with gracilis tendon, n = 2 triple semitendinosus graft, n = 1 M. rectus tendon.

Two patients suffered from additional meniscal tears, which were sutured during the same procedure. Mean preoperative IKDC score was 67.4 (39.1–87.4). Mean IKDC score improved in 3 patients from a mean of 52.9–84.3 6 months after operation, in one patient from 74.7 to 80.5, in one patient from 78.2 to 94.3 18 months after the operation and in 2 patients from average 73.6 to 97.7 2 years postoperatively. There were no complications. All patients had a negative Lachman test after surgery. Postoperative radiographs revealed no damage to the physes.

***Discussion*** All-inside all-epiphyseal anatomic single-bundle ACL reconstruction in Tanner stage 1 and 2 patients with open physis has a favorable outcome with a low rate of complications. The aimed follow-up of 2 years is still incomplete for 9 patients, but preliminary results are very promising at 6, 18 and 24 months after surgery.

### Neuroorthopedics: ePoster

***Neuroorthopedics: ePoster VKO-166***

#### *Maximum Deformity of Hip and Knee Joint in a 15 Year-Old Refugee Child from Eritrea with Cerebral Palsy*

H. Lengnick[1], J. Cip[2], M. Olach[2], K. Studer[2], E. Payne[2], H. Klima[2]

[1]Ostschweizer Kinderspital St.Gallen, Kinderorthopädie, St.Gallen, Schweiz, [2]Ostschweizer Kinderspital, Kinderorthopädie, Claudiusstrasse 6, 9006 St.Gallen, Kinderorthopädie, St.Gallen, Schweiz

***Question*** We present the case of a 15-year old refugee child from Eritrea suffering from bilateral spastic cerebralparesis (CP) with gross motor function classification system (GMFCS) level V. Due to hydrocephalus he got at the age of 14 months a ventriculo-peritoneal (VP) shunt supply at his homeland. In his past medical history, massive macrocrany developed over the years without further medical management.

***Methodology*** Living since September 2014 in Switzerland he presented a few months later the very first time in our clinic due to pain exacerbation at the right hip during transfer and personal hygiene. The clinical examination showed flexion contractures at both hips of about 90°, abduction of only 10° combined with nullified rotation in terms of ankylosis. Flexion contractures of both knees by 110° were observed, while both feet showed pes calcaneovalgus deformity.

Trunc stability was not existent due to the massive makrocrany and the not controllable weight of his head. X-ray verified high luxation of both hips leading to pelvic obliquity. As consequence of this deformity a leftconvex lumbal scoliosis had developed. Use of the upper extremity was reduced but possible because of flexion contractures of the elbow and wrist on both sides. While the deformities of upper and lower extremity were immobilizing, he showed a positive cognitive condition with facilities in German and English language.

***Result*** We performed extraction of the femur head on the right side as a first treatment step to reduce pain situation. Management of transfer and daily personal hygiene were followed with storage devices and wheel chair. Further examinations for possibility for operative stabilization of the spine by respecting the VP-shunt were planed by spine and paediatric surgeons.

***Conclusion*** We present a tetraspastic CP-patient with the maximum form of contractures of the hip and knee joints. Showing painful high hip luxation and lumbal scoliosis at the age of 15, this is a negative example for natural history development, if no medical measures are undergone to attend these patients until adolescence and beyond.

On the other hand it is an example for the necessity of medical help and treatment for refugee people coming to Europe. The possibility to help and to take responsibility for these human beings should be part of physicians daily work away from political discussions about refugee upper limit.

### Neuroorthopedics: Lecture

***Neuroorthopedics: Lecture VKO-174***

#### *Development of a Classification System for Wheelchair Maintenance and Seat Positioning for Patients with Neuro-Muscular Diseases and Dysmorphia of Skeleton*

S. Kappl[1], T. Haßlberger[1], E. Sturm[2], H. Bangert[2], S. Nader[1]
[1]Schön Klinik, Klinik für Kinderorthopädie, Vogtareuth, Deutschland, [2]Spörer Gesundheitsgruppe, Vogtareuth, Deutschland

***Question*** Taking care of above named collective of patients is difficult as the disease pattern is so different. Hence, the demand varies. The aim is to develop a classification system for seat and wheelchair maintenance which is transparent, independent from manufacturer and clinically based.

***Methodology*** An appendix considers the patient́s satisfaction and that of the relatives. The system combines 10 clinical tests consisting of 1–5 points each. The minimum to be reached is 10 points, the maximum 50 points. 5 categories are distinguished. Category I is independent from scoring (sports wheelchair). Categories II–V are divided into standard active wheelchair to special constructions just for transfer.

58 patients (38/20 m/f), mean age 10.3 years, range of time 12 months. The test of the classification system was performed by 2 teams. Team I: All patients known by person. Team II: Patients rather unknown. The team splitting was done by the last two reports, conclusions were allowed.

***Result*** 20 complete documents, Team I and II showed 85 % of correspondence with a difference of ±1 point in total. Interrate reliability W = 0.87. Missing personal details were adjusted (+1P) showing a difference of ± 1 Point in 57 %. The mean score difference without missing details is 0.85, with missing details 5.62 points. A significant difference is thus proofed ($p < 0.0001$).

Within the full-detail-score a correspondence of 85 % is found, within the missing-detail-score 43 %. After adjustment (+1P) the correspondence is 67 %. The mean difference of the categories within the full-detail-score is 0.15, within the missing detail-score 0.92 categories. Poor evaluation concerned the power (45 %), the contraction (24 %) and the movement (17 %) of the upper extremity.
***Conclusion*** The classification system is an applied method. A clinical based, manufacturer independent maintenance is possible. The system helps in orientation. The quality of documentation must be improved to gain a standard, thus making it comprehensible. The affect of environment must be taken into account better than now. Further checks on the classification system with different disease patterns are necessary as well as checking the quality of maintenance.

***Neuroorthopedics: Lecture VKO-139***

### *Gastrocnemius Function in Crouch Gait: a Direct Muscle Imaging Study in Children with Bilateral Spastic Cerebral Palsy*

M. Hösl[1,2], A. Keymer[3], H. Böhm[1], A. Arampatzis[2], L. Döderlein[1]

[1]Behandlungszentrum Aschau GmbH, Orthopädische Kinderklinik, Aschau im Chiemgau, Deutschland, [2]Humboldt-Universität zu Berlin, Abteilung Trainings- und Bewegungswissenschaften, Berlin, Deutschland, [3]Technische Universität München, Biomechanik im Sport, München, Deutschland

***Question*** Spastic crouch gait is inefficient and naturally deteriorates. While crouch seems primarily a deformity of the knee, shortness of the gastrocnemius might promote crouch, since the bi-articular plantarflexors could steel motion from the knee [1]. Besides, it is postulated that during crouch, spastic gastrocnemius muscle fibres overstretch which potentially causes muscle fibrosis and contracture [2, 3]. Our aim was to study the gastrocnemius contractile behavior directly during crouch.
***Methodology*** 15 children with BSCP (GMFCS I + II, no pre-OP, no knee contracture) and 17 typically developing (TD) peers (7–16 years) participated. BSCP walked in mild-moderate crouch (mean knee flexion > 1SD from TD). They were analyzed at rest and while walking on a treadmill. Leg kinematics were measured with Vicon, shank muscle activity with surface EMG and ultrasound videos visualized the med. Gastrocnemius (MG). We tracked fascicles (fibre bundles) and calculated tendonious tissue length to extract concentric and eccentric excursions as well as max. length during stance. All parameters were normalized on resting values. Children also underwent PF-force tests using hand-held dynamometry and were clinically examined.
***Result*** Passive DE in BSCP was constrained (1° ± 8° vs. 15° ± 4°) with a highly pos. Silferskiold sign. PF-force was 28 % less. During rest, spastic MG was 19 % thinner and fascicles 17 % shorter (all $p < 0.003$). The tendon tended to be longer (+2 %, $p = 0.081$). BSCP walked with similar dorsiflexion, but considerably bent knees. They reached less max. fascicle length (−12 %, $p = 0.005$) with similar eccentric ($p = 0.582$) and 42 % less concentric excursion ($p < 0.001$). The tendonious tissue experienced 5 % less pull by the muscle ($p = 0.005$). Correlations showed that BSCP with smaller fascicles displayed larger knee flexion during gait ($r = -.69$, $p < 0.001$). Neither clinical knee extensor strength or shorter hamstrings were sign. correlated (both $p > 0.19$).
***Conclusion*** BSCP had short, weak and atrophied MG. The hypothesis that fascicles overstretch seems invalid [2] but shortness of MG fascicles induced crouch! In high-functioning ambulators with BSCP, flexed knees could be a functional adjustment to use short MG fascicles in a muscle-joint configuration where they are able to produce their largest active force. Success of knee-related surgeries against crouch may be limited if MG fascicles lack simultaneous growth.

***References***
[1] Maas et al., J Electromyo Kines, 2015
[2] Pitcher et al., Muscle Nerve, 2014
[3] Lorenzo et al., Med Hypotheses, 2015

***Neuroorthopedics: Lecture VKO-176***

### *Hereditary Sensory Autonomic Neuropathy Type IV: An Attempt to Elaborate a Therapeutic Concept Based on Literature and Clinical Experiences*

A. Kellermann, A. Schraml

Cnopfsche Kinderklinik, kinderorthopädische Chirurgie, Nürnberg, Deutschland

***Question*** The treatment of children suffering from the rare autosomal recessive disorder of hereditary sensory autonomic neuropathy type IV, challenges us as doctors repeatedly with major problems.
***Objectives*** The aim of this work is to verify if the development of a general therapeutic concept is eligible. In particular, emphasis will be put to the orthopedic field.
***Methodology*** A trial to create a therapy concept was based on 31 published case reports by 19 different authors, five clinical reports from pediatric orthopedic centers and three case reports from a personal clinical experience.
***Result*** One of the main reasons for initial medical consultation is a high fever of unknown origin, which can be life threatening. For further treatment strategic focus is placed on the treatment of pressure ulcers, the consequences of mutilation, such as osteolysis, infections and even amputations. The mobility is jeopardized by a high number of fractures, joint luxations and Charcot joints. The healing process of these injuries is aggravated by increased incidence of nonunions, hypertrophic callus formation, wound healing disorders and implant dislocations.
***Conclusion*** The development of a general therapeutic concept cannot be established. The main focus of therapy should be placed on fracture prophylaxis by means of full contact orthosis and behavioral therapy, as well as the guidance of parents to be alert concerning early detection of infections, pressure ulcers and fractures. The indication for surgical treatment or corrective osteotomy must be made cautiously.

***Neuroorthopedics: Lecture VKO-183***

### *Hip Surveillance in Cerebral Palsy: Lessons Learned?*

S. Lebek[1], C. Langer[2], K. von Au[2], K. Baumgart[3], J. Funk[4]

[1]Charité- Universitätsmedizin Berlin, CMSC, Kinder- und Neuroorthopedics, Berlin, Deutschland, [2]Charité - Universitätsmedizin Berlin, Sozialpädiatrisches Zentrum für chronisch kranke Kinder, Berlin, Deutschland, [3]Helios Klinikun Emil von Behring, Kinderorthopädie, Berlin, Deutschland, [4]Charité- Universitätsmedizin Berlin, CMSC, Kinder- und Neuroorthopedics, Berlin, Deutschland, Berlin, Deutschland

***Question*** For 20 years hip surveillance programmes for children with cerebral palsy (CP) have been successfully used in several countries. Paediatrc orthopaedic surgeons and neuropaediatricians have popularized the information in Germany. We wanted to know if the knowledge of such programmes would suffice to prevent hip dislocation in children with CP treated in a multidisciplinary clinic.

***Methodology*** We performed an audit in order to assess the status of hip surveillance in children with cerebral palsy attending our institution from March 2014 to April 2015.
***Result*** Of 527 patients coded as cerebral palsy 457 suffered from CP by definition. 30 children suffered from unilateral CP, 153 from bilateral spastic CP, one from ataxic CP, 18 dyskinetic CP and in 30 patients a dominant symptom was not coded.
Only 247 patient older than 2 years of age received at least one pelvis X-ray between their first and last consultation with the neuropaediatrician. The functional levels were as follows: GMFCS I n = 36, II n = 50, III n = 63, IV n = 33 and V n = 65 [mean age 9.6 years (2–18 years); 147 male, 100 female].
Of the 247 patients with X-ray, 51 X-rays showed a Reimers migration percentage (MP) of >50 %, 51 a MP 31 to 49 % and the rest <30 %.
***Conclusion*** We analyzed these rather disillusioning results, the exterior differences to functioning hip surveillance systems as well as the interior problems.
There are several ways to improve the results. The ideal way is the implementation of a nationwide screening programme, but this will need to be organized and will cost money.
Another possibility is to organize a "sensible" screening for the children at risk (with GMFCS III–V) according to F. Miller.
Our solution was to communicate the results within our institution and to start a special clinic only for hip surveillance in CP according to the already communicated X-ray standards and clinical examinations.
As long as there is no nationwide screening programme any paediatric orthopaedic surgeon concerned with the care of CP children should act according to the known rules of hip surveillance.

***Neuroorthopedics: Lecture VKO-126***

### *Long-Term Results of Single-Event Multilevel Surgery in Children with Spastic Hemiplegia*

M. Svehlik, C. Schranz, G. Steinwender, M. Sperl, T. Kraus

Univ. Klinik für Kinderchirurgie, Abt. für Kinderorthopädie, Graz, Österreich

***Question*** There is solid evidence that single-event multilevel surgery (SEMLS) may improve gait in children with bilateral cerebral palsy and that those improvements can be maintained in long term. However, there is no evidence on long-term outcomes of SEMLS in children with unilateral spastic cerebral palsy.
***Methodology*** This is a retrospective analysis of clinical and gait analysis data. We included children with unilateral spastic cerebral palsy with the Gross Motor Function Classification System Level I to III preoperatively and a minimal follow-up of 5 years. All children were evaluated using gait analysis before, 1, 3–5 years and at the most recent examination after SEMLS. The Gait Profile Score was used to evaluate the postoperative changes. The four time points were compared using a repeated measures analysis of variance with a pairwise comparison (SPSS Inc., Chicago, IL, USA).
***Result*** The average GPS improvement of 2.2° is well above the minimal clinical important difference of 1.6°. This shows that the postoperative gait improvement was clinically meaningful. Moreover, no significant deterioration occurred during the 10 years of follow-up even if some fine-tuning procedures were needed. The results are in accordance with studies on children with bilateral cerebral palsy.
***Conclusion*** The study indicates that SEMLS are beneficial and postoperative gait improvements clinically meaningful in children with unilateral cerebral palsy even in the long term run.

***Neuroorthopedics: Lecture VKO-155***

### *Long-Term Therapy with Intrathecal Baclofen Improves Life Quality in Children with Severe Spastic Cerebral Palsy*

T. Kraus[1], K. Gegenleithner[1], M. Svehlik[1], M. Sperl[1], G. Singer[2], G. Steinwender[1]

[1]Medizinische Universität Graz, Kinderorthopädie, Graz, Österreich, [2]Medizinische Universität Graz, Kinderchirurgie, Graz, Österreich

***Question*** Children with severe spastic cerebral palsy (CP) are highly limited in daily life activities causing a reduced quality of life (QoL). This is partly due to an increased muscle tone causing pain and contractures. Continuous intrathecal infusion of baclofen (ITB) reduces the spasticity of affected patients. The hypothesis of the present study was that ITB leads to a significant improvement of QoL in non-ambulant children with CP.
***Methodology*** 13 patients (10 male, 3 female, mean age 14 years) were included. Mean time between pump implantation and follow up was 60 months (range 12–100). QoL was assessed before and after baclofen pump implantation using standardized questionnaires (CP CHILD, KINDL). Spasticity was evaluated using the modified Ashworth Scale (MAS) at the two time points.
***Result*** QoL improved from pre-implantation to follow up. MAS markedly decreased from 3.8 to 1.7. All interviewed participants indicated, that their expectations had been met and that they would choose ITB treatment again.
***Conclusion*** Intrathecal treatment of Baclofen is an excellent method for spasticity management in children with severe cerebral palsy. Quality of life sustainably improves; parents' satisfaction is high and the level of spasticity decreases. Therefore, Baclofen treatment can be highly recommended in non-ambulant children with CP suffering from spasticity.

***Neuroorthopedics: Lecture VKO-161***

### *Possibilities and Limitations of the Newest Generation of Video Based 3D Gait Analysis Systems*

V. Hirschmann[1], P. Russ[2], L. Becker[2], W. Strobl[1]

[1]Krankenhaus Rummelsberg, Schwarzenbruck, Deutschland, [2]Simi Motion, Unterschleißheim, Deutschland

***Question*** The newest generation of video based 3D gait analysis systems provides the opportunity to operate 3D movement analysis markerless, markerbased and hybrid. Markerless tracking is seen as a potential technology to make movement analysis simpler, quicker and less error-prone through wrong marker placement. This case study evaluates the accuracy of markerless and hybrid tracking against traditional marker based tracking. The aim of this study is to research if markerless tracking is comparable insertable for healthy and patients with cerebral palsy.
***Methodology*** Patients with cerebral palsy and healthy persons without any limitations will be recruited. The patients are asked to walk along a 12 m gait walk. The patients will be marked with the marker model for lower extremities. Markerless data, hybrid data and marker data will be recorded at the same time using 8 cameras (2.0MP@100 Hz). 3D Marker data will be obtained using Simi Motion 3D (<0.01 mm mean failure), joint angels will be computed using Simi Motion Inverse Kinematics module. Markerless data will be processed with Simi Shape. Hybrid data will be calculated with Simi Shape using different marker combinations to assist silhouette tracking. The results will be compared using the spearman correlation coefficient.
***Result*** Only for ankle eversion/inversion inaccurate results with markerless tracking will be expected. Furthermore a high correlation between markerless and markerbased tracking of hip flexion/extension and ankle abduction/adduction and a very high correlation of hip rotation and abduction/adduction, knee flexion/extension and ankle platar-/dorsiflexion will be expected. It can be assumed that with hybrid tracking all measured joint angles correlate very high with markerbased data.
***Conclusion*** This study shows that markerless tracking can achieve good results for most joint angles. Some angles like ankle eversion/inversion can't measured accurately with markerless tracking. However to achieve very good results in all joint angles hybrid tracking with few additional markers to support silhouette tracking is necessary. This is important for segments where geometric appearance barely changes during rotation. This study shows that it will be possible to reduce the markers, thereby safe time and reduce the potential for errors through wrong marker placement. A pure markerless system will also be applicable for basic questions in clinical gait analysis.

***Neuroorthopedics: Lecture VKO-151***

### *Predictors for Pelvic Anterior Tilt after Patella Tendon Shortening in Children with Cerebral Palsy*

H. Böhm[1], M. Hösl[2], L. Döderlein[2]

[1]Orthopädische Kinderklinik, Ganglabor, Aschau im Chiemgau, Deutschland, [2]Orthopädische Kinderklinik, Kinderorthopädie, Aschau, Deutschland

***Question*** Patellar tendon shortening procedure within single event multilevel surgeries was shown to improve crouch gait in Cerebral Palsy (CP) patients [1]. However, one of the drawbacks associated to patella tendon shortening may be increased pelvic anterior tilt with compensatory lumbar lordosis [1]. Since the compensatory lumbar lordosis leads to backpain and impairs the walking ability, the aim of this study was to show what distinguished patients who showed excessive anterior tilt after patellar tendon shortening.
***Methodology*** Thirtytwo patients with spastic cerebral palsy GMFCS I (4) and II (28) mean age 13.4 (SD = 3.3) years were included. All 49 limbs that were operated received patella tendon shortenings within multilevel surgery. Instrumented gait analysis and clinical testing was performed pre- and 24.1 (SD = 3.5) months postoperatively. Stepwise multilinear regression of the response value mean pelvic tilt during stance phase was performed from preoperative clinical and gait measurements.
***Result*** The mean improvement of the knee extension during walking was 13° (SD = 4°). 40 % of the legs showed more than 5° increased pelvic anterior tilt during walking postoperatively. Reduced walking speed together with reduced hip extension power during stance phase together explained 38 % of the increase in pelvic anterior tilt. Hip contracture or hip extension during walking did not show any predictive significance. Multilevel procedures in addition to the index procedures relevant for pelvic motion were 32 knee extension osteotomies, 15 rectus recessions 7 psoas and 3 knee flexor lengthenings. Those legs with considerable increase in pelvic anterior tilt had 8/32 extension osteotomies, 5/15 rectus recessions 0/7 psoas and 1/3 knee flexor lengthenings.
***Conclusion*** In conclusion patients with slower walking speed and less hip extension power during walking pre-op were at higher risk of anterior pelvic tilt post-op. These patients may have insufficient control and strength of hip extensors during walking to adapt to the situation of improved knee extension during walking. The group with less anterior tilt had more frequent psoas lengthenings and proximal rectus recession procedures. These procedures may reduce the tension at the anterior hip muscles to allow a more extended hip following surgery.

***References***
[1] Sossai et al. Gait Posture 2015; 41:658–65

***Neuroorthopedics: Lecture VKO-172***

### *The Elbow in Arthrogryposis Multiplex Congenita*

C.U. Dussa, A. Peterarndel, L. Döderlein

Orthopädische Kinderklinik, Kinderorthopaedie, Aschau i. Chiemgau, Deutschland

***Question*** Joint contractures are hallmark of Arthrogryposis multiplex congenita. The aim of the study was to look at the different patterns of presentation of Elbow in Arthrogryposis multiplex congenital.
***Methodology*** In all these cases a standard examination form was used to measure the range of movement of elbow, muscle strength, degree of contracnture. A photo and video documentation was performed in every case. Exclusion criteria were lack of standardized documentation, previous surgery, Pterygeum Syndromes, Associated plexus injury.
***Result*** Until December 2014, 96 Patients with index diagnosis Arthrogryposis multiplex congenital were seen and documented in the Ergotherapy. Using the exclusion criteria 36 Patients were excluded from the study. Therefore the study comprised of 60 Patients with 119 Elbows. The age group ranged from 3 to 23 years with 28 males and 32 females. Symmetrical involvement was seen in 37 Patients and asymmetrical in 23 Patients.
Based on the involvement, the elbows could be classified into 4 groups. Group A: (1) Flexion contracture <10°, (2) Active flexion and extension, (3) <10° difference between active and passive movements. Group B: (1) Presence of flexion contracture >20°, (2) B+ —Passive = Active flexion ≥90° (Biceps MRC 4/5), (3) B——Passive flexion ≥90°, Active flexion <45° (Biceps MRC <3/5). Group C: (1) Full elbow extension, (2) Active Triceps (MRC >4/5), (3) No active flexion or passive & active flexion <30° and 4. C+—Active flexion <30° & passive flexion <80°. Group D: (1) Weak or absent Biceps and weak Triceps, (2) No or flexion contracture <10°, (3) Passive flexion and extension full (near).
About 50 % of the elbows belonged to group A, and therefore had normal function. The uncommon form of elbow involvement is Group D with passive near full range of movement with no active function.
***Conclusion*** In the literature the involvement is only described as flexion and extension types. In our study we have noticed several other naturally occurring forms of elbow involvement with as we noticed can be group into 4 types. The study throws new information on the involvement of the elbow and therefore gives information on about what percentage of elbows that may need treatment of any kind. The compensatory and adaptive movements are also different in each group except group A.

***Neuroorthopedics: Lecture VKO-199***

### *The Results of Preoperative Halo-Gravity Traction for the Treatment of Severe Spinal Deformity in Children*

B. Erdinger, K. Mladenov, U. von Deimling

Asklepios Kinderklinik, Kinderorthopädie, Sankt Augustin, Deutschland

***Question*** Halo gravity traction has been used preoperatively for the correction of severe spinal deformity in children with good results. However the reported overall complication rate remains high and varies between 19 and 33 %.
***Purpose*** To assess the efficacy and safety of our treatment protocol for preoperative halo gravity traction in children with severe spinal deformity with respect to radiographic outcomes and clinical complications.
***Methodology*** Single centre, retrospective study from 2011 to 2015 of 22 paediatric patients with severe, rigid scoliosis and/or kyphosis of more than 100°. The deformity was of neuromuscular (n = 9), syndromic (n = 5), idiopathic (n = 4) and dysplastic (n = 4) origin. The patients underwent preoperative, gradual, transferable, continuous halo gravity traction (bed, wheelchair, walker) of up to 40 % body weight, applied for an average of 6 weeks according to our established treatment protocol. Data were analysed with emphasis on complications. Deformity correction was evaluated using serial, standing X-rays (pre-treatment, at completion of traction and after surgery).
***Result*** 11 patients underwent after completion of traction spinal fusion and 11 were treated with a "non-fusion"-technique. In no case was an anterior release necessary.
Cobb Angle before traction showed a variation range between 100° and 174°. After completion of traction radiographic outcomes showed a mean improvement of 33° (range 21°–57°) and an additional 25° (range 2°–53°) improvement after surgical intervention. The total mean improvement amounts to 59° (range 34°–101°). The share in overall Cobb Angle improvement through traction was considerably higher in patients with kyphosis (70 %) than in patients with scoliosis (60 %). There were no neurologic complications. In only 3 Patients minimal pin irritation occurred during traction which healed with local measures.
***Conclusion*** Halo gravity traction is a safe method for gradual correction of severe spinal deformity in children. Complication rate can be significantly reduced using standardised treatment protocol.

## Foot Deformities: ePoster

***Foot deformities: ePoster VKO-111***

### *Results After Percutaneous Muscle Release of a Flexible Plat Foot in a 8 year old Child with Cerebral Palsy*

V. Gattung, P. Bernius

Schön Klinik München Harlaching, Zentrum für Kinder- und Neuroorthopedics, München, Deutschland

***Question*** In patients with cerebral palsy 25 % suffer from a planovalgus foot (1). Early correction is recommended to prevent an aggravation and to improve function (1).
In literature several methods are described for mobile children with cerebral palsy, as treatment with botulinum Toxin type A (2), (3), extraarticular subtalar arthrodesis (4).
Immobile children with cerebral palsy (GMFCS 5) also need a stable foot situation to allow standing, which is essential to avoid pulmonary dysfunction, to improve hip developement and to participate social live.
In worse cases of spasticity we often see severe foot deformity in young aged patients. Botulinum toxin therapy has often poor results in severe foot deformity, whereas arthrodesis decreases joint function. Therefore, we prefer soft tissue balancing surgery as a good alternative to improve foot situation.
***Methodology*** We did a percutaneous release in an 8 year old male child with cerebral palsy GMFCS 5 and a severe but flexible planovalgus foot. Because of pressure marks it was not longer possible to wear orthosis. The parents did not want arthrodesis, botulinum toxin would not be not effective. We did a percutaneous myofasciotomy of M. gastrocnemius, M. peroneus and M. extensor digitorum longus to reduce muscle tension which is responsible for foot deformity. Additionally, we did a temporary transfixation of the talocalcanear and subtalar joint with a K-wire for 3 months. We did radiographic controls before and after operation and measured the talocalcaneal angle in both radiographic planes as a degree of talonavicular joint subluxation.
***Result*** Preoperatively we measured on the right foot a TC angle of 50° in the lateral view, which was improved to 37° postoperatively. The TC angle was 41° in the lateral view of the left foot and improved to 34° postoperatively. The TC angle in the frontal view was 53° preoperatively and 30° postoperatively for the right foot and 31° to 25° for the right foot.
***Conclusion*** Percutaneous muscle release in a flexible planovalgus foot in young patients with cerebral palsy without gait function can approve or even stabilise foot situation with a good clinical result. Furthermore, foot position now allowed orthosis treatment.
Further observations will be needed to examine the future development in foot function and if the long-term result will show enough stability of the feet for weight bearing purposes.
Even if arthrodesis is required later, we can at least gain time.

***References***
[1] Molayem, Persiani, Marcovici, Rosi, Calistri, Villani, (2009), Complications following correction of the planovalgus foot in cerebral palsy by arthroereisis
[2] Kurenkova, Dutikova, Nikitin, Artemenko, (2010), Foot deformity in children with spastic forms of cerebral palsy: the treatment with botulinum toxin type A (dysport)
[3] Sätilä, Huhtala, (2010), Botulinum toxin type A injections for treatment of spastic equinus in cerebral palsy: a secondary analysis of factors predictive of favorable response
[4] Yoon, Park, Roh, Park, Chi, Kim, (2010), Extraarticular subtalar arthrodesis for pes planovalgus: an interim result of 50 feet in patients with spastic diplegia

***Foot deformities: ePoster VKO-187***

### *Wait and Watch vs. Early Operative Therapy in an Extremely Low Birth Weight Newborn with Necrotic Feet and Fingers*

M. Vogt, M. Rogalski, M. Deja, F. Sitterlee, D. Kendoff

Helios Klinikum Berlin-Buch, Zentrum f. Orthopädie und Unfallchirurgie/Kinderorthopädie, Berlin, Deutschland

***Question*** The right treatment of early newborn children with gangrenes of the feet and fingers means a big challenge of taking the right treatment decisions since there are not many similar cases reported in the literature. We want to present the case of a 7 years old mentally retarded former preterm born boy (birth weight 480 g, GA: 27th week of pregnancy) who developed vasospastic peripheral gangrenes of all extremities in the first days after birth.
Our goal was to offer the right therapy to the ELBW newborn child concerning the necrotic upper and lower extremities in the knowledge of other complications such as respiratory distress syndrome and intraventricular hemorrhage
***Methodology*** We chose the therapy of wait and watch and well thought operative procedure. The necessary surgery was combined soft tissue balancing and osseous adaptation of the right foot at walking age as well as orthotic and physiotherapeutic management for symptoms of mild cerebral palsy as sequelae of IVH.
***Result*** After 7 years the boy is able to walk with orthoses and can live a life of at least independent mobility (GMFCS II-level).
***Conclusion*** The alternative treatment of early operative procedure with amputation of the lower leg would have meant a dependency on wheelchair since there would have been no possibility to learn how to walk without any response of proprioceptive reflexes. Therefore wait and watch treatment before surgery and the interaction of other disciplines as paediatric surgery, NICU-treatment, dermatology and angiology was the right procedure in this difficult case of an extremely low birth weight newborn with gangrenes of feet and hands.

### Foot deformities: Lecture

***Foot deformities: Lecture VKO-196***

#### *Changes in Foot Motion and Plantar Pressure After Tibialis Anterior Tendon Transfer for Clubfoot Recurrence*

G. Mindler[1], A. Kranzl[2], C. Radler[1]

[1]Orthopädisches Spital Speising, Abteilung für Kinderorthopädie und Fußchirurgie, Wien, Österreich, [2]Orthopädisches Spital Wien Speising, Labor für Gang- und Bewegungsanalyse, Wien, Österreich

***Question*** The tibialis anterior tendon transfer (TATT) is an effective joint-sparing operation in children with dynamic clubfoot recurrence. However, only few studies used pedobarography or gait analysis to evaluate outcome after TATT. Our aim was to evaluate changes in pedobarography and gait analysis including a foot model after TATT. Preliminary results of this prospective study are presented.
***Methodology*** Patients older than 3 years with idiopathic clubfoot treated with the Ponseti method at our center and with a TATT were included. Exclusion criteria were prior bony or joint invasive clubfoot surgery and associated syndrome or neurological disease. The indication for TATT was dynamic clubfoot recurrence with foot adduction and supination during swing. Postoperative gait analysis and pedobarography were performed 6 months after surgery and compared to the preoperative data. A total of 63 children underwent tibialis anterior tendon transfer between 2011 and 2015; 30 patients met the inclusion criteria.
***Result*** A total of 10 children (6 bilateral, 4 unilateral) with 16 clubfeet with a mean age at time of surgery of 7 (5–9) years were evaluated. Additional surgical procedures were Achilles tenotomy in 6 feet, plantar fasciotomy in 2 feet and abductor hallucis release in one foot. Gait analysis showed significant increase of ankle dorsiflexion during the whole gait cycle. The mean range of motion of the hindfoot in relation to the tibia (dorsiflexion/plantarflexion) increased significantly. Furthermore, the Oxford foot model showed decreased forefoot adduction and supination. The gait deviation index showed significant improve of the overall gait pattern after TATT. Pedobarography showed decreased maximal force, force time integrals at the lateral aspect of the midfoot.
***Conclusion*** Gait analysis with the Oxford foot model and pedobarography showed positive effects of TATT on foot motion and plantar pressure distribution in children with clubfoot recurrence. However, further evaluations of larger cohorts with a longer follow up should be obtained.

***Foot deformities: Lecture VKO-184***

#### *Early Complications After Subtalar Arthroereisis in the Treatment of the Juvenile Flatfoot*

D. Dovi-Akue, K. Kuminack, H. Grünewald, N.P. Südkamp, A. El Tayeh

Klinik für Orthopädie und Unfallchirurgie, Universitätsklinikum, Freiburg, Deutschland

***Question*** The subtalar arthroereisis is an increasingly performed operation for the correction of the juvenile flatfoot. Indication is the symptomatic flexible, non-rigid, flatfoot with hindfoot valgus. We performed a retrospective study to identify possible early complications.
***Methodology*** All patients that were operated for flexible juvenile flatfoot deformity using the Arthrex® ProStop® screw during the period of 06/2013–06/2015 were included in the study. Follow-up was reviewed upon pain, wound infection, dislocation of the screws and necessity of a secondary operative intervention.
***Result*** 29 patients were included into the study, 13 boys and 16 girls aged 9–18 years (mean 13.2 years, standard deviation ±1.88). Follow up was performed upon 44 operations including 30 bilateral interventions.
With a mean follow-up time of 10 months we found no intraoperative complications or wound infections. Nine flatfoot corrections with subtalar arthroereisis (20.5 %) were in need of a secondary operation. Two patients (9 %) with bilateral arthroereisis required replacement of the ProStop® screw with larger implants due to growth related implant loosening. Four screws (9 %) had to be removed due to persisting load-dependent pain including two overcorrections and one dislocation toward the posterior talocalcanear joint. One screw (2.2 %) had to be reimplanted due to lateral dislocation. Four patients described minor load-dependent pain (6 feet/13.6 %).
***Conclusion*** Persisting pain and secondary dislocation—occasional or growth related—are the most common early complications in flatfoot correction by subtalar arthroereisis using ProStop® screws.

***Foot deformities: Lecture VKO-170***

#### Early Treatment of Juvenile Hallux Valgus

K. Studer, J. Cip, M. Olach, H. Lengnick, E. Payne, H. Klima

Ostschweizer Kinderspital, Abteilung Kinderorthopädie, St. Gallen, Schweiz

***Question*** Juvenile hallux valgus (JHV) deformity is described as a varus deviation of the first metatarsal and valgus deviation of the first proximal phalanx, occurring in a child who is skeletally immature. Juvenile hallux deformity is not common in childhood and different therapeutic strategies are possible. An elegant method of correction is to influence growth by a permanent lateral hemiepiphyseodesis of the first metatarsal bone.

***Methodology*** We retrospectively reviewed all patients operated from 2012 to 2014 with the diagnosis of a juvenile hallux valgus deformity at our institution. Included in our study were all patients with the operative technique of a permanent lateral hemiepiphyseodesis of the first metatarsal physis combined with a soft tissue correction (modified McBride procedure). Postoperatively a soft bandage was applied redressing the first toe in a varus position for 4 weeks day and night, going on during night for another year. Patients with additional necessary operations and/or a main deformity of a hallux valgus interphalangeus were excluded. We evaluated radiographs at the initial visit, 6 weeks postoperatively and 1 year postoperatively. The intermetatarsal angle (IM), hallux valgus angle (HV) and the distal metatarsal articular angle (DMAA) were obtained in degrees (°).

***Result*** Twenty-two patients could be included affecting 12 bilateral, 5 left and 3 right feet. Mean age at operation was 11 years 2 months (9–13 years). The mean IM was 13.25° (8°–19°). The mean HV angle was 22.2° (8°–39°). The mean length of MT I was 56.7 mm (46.3–64.9 mm). Median DMAA was 12.1° (6.4°–22.1°).

One year postoperatively we found the following results: Mean IM angle was 10.78° (mean 6°–18°). Mean HV angle was 15.2° (7°–33°). Mean DMAA was 7.3° (2°–25°). Mean length of MT I was 57.2 mm (44.7–65.5 mm).

90 % of our patients showed a good correction of the hallux valgus deformity. In two patients the operation was unsuccessful due to advanced age (one patient), respectively another patient did not wear the bandage postoperatively.

***Conclusion*** Permanent lateral hemiepiphyseodesis of the first metatarsal physis with soft tissue release is a very good technique correcting a juvenile hallux valgus deformity.

The success rate is high provided that the child has enough foot growth left.

Because of this fact, we recommend that maximal age should be 10–11 years in girls and 11–12 years in boys at time of operation. Postoperative wear of correcting bandage is an absolute necessity.

***Foot deformities: Lecture VKO-173***

### *Serial Ankle Casts for Patients with Idiopathic Toe Walking: Effects on Functional Gait Parameters*

C. Druschel[1], G. Rockstroh[2], J. Mehrholz[3], F. Thielemann[4]

[1]Uniklinikum Dresden, UniversitätsCentrums für Orthopädie und Unfallchirurgie, Dresden, Deutschland, [2]Klinik für Orthopädie, Biomechanik, Kreischa, Deutschland, [3]Private Europäische Medizinische Akademie, Kreischa, Deutschland, [4]UniversitätsCentrum für Orthopädie und Unfallchirurgie, Abteilung für Kinderorthopädie, Dresden, Deutschland

***Question*** Toe walking is one of the most prevalent gait deviations. Idiopathic toe walking (ITW) is the diagnosis of toe walking without evidence of an underlying medical condition. As long-term consequence ITW leads to a reduced ankle range of motion and therefore often to a poor sport performance. Reported ITW treatment have included serial casting, Botulinum toxin type A or surgery. However, non-surgical therapy is little described with minimal quantitative pre/postinterventional analysis. The aim of this study was to investigate the functional effects on gait parameters of serial ankle casts for patients with idiopathic toe walking.

***Methodology*** Within a prospective trial we included 10 patients with ITW and 10 healthy matched children (aged between 5 and 15 years). Children with ITW underwent serial casting to stretch the plantarflexors: two times 14 days walking plaster casts set at maximum available ankle dorsiflexion. Both groups were assessed using a functional gait analysis before/after serial casting and during a 6 month follow up visit. Obtained gait parameters: force of heel strike, upper ankle joint-angle, angle between sole of foot and floor plus the compound action potential of the left and right gastrocnemius muscles medial head, displacements of the knee joint in the sagittal plane and the variation of the centre of gravity.

***Result*** Significant improvement in functional gait parameters was achieved shortly after casting as well as during follow up. The normalized plantar heel force increased from 5 to 79 %. Also upper ankle joint-angle and base angle demonstrated significant changes. Normalized compound action potentials of the M. gastrocnemius medial head were reduced by 70 %. All of these parameters demonstrated no significant difference at follow up compared to the healthy control group.

***Conclusion*** Compared to the literature we have proven serial casting to achieve significant long-term improvement concerning functional kinematic parameters in ITW. Muscular detonization with lengthening of the ankle plantarflexors led to a persistent increased active ankle dorsiflexion. This positive cast effect is emphazised by the absence of any significant differences in the gait analysis between the ITW group and healthy children 6 month after treatment. Furthermore, it was found that the non-invasive serial cast treatment and non-invasive diagnostic procedures were inoffensive and largely free from discomfort for the patients.

***Foot deformities: Lecture VKO-148***

### *Surgical Treatment of Talocalcaneal Coalition in Childhood and Adolescence: Experience and Unfavourable Results in 80 Cases During a 13-Year-Period*

J. Hamel, M. Nell

Zentrum für Orthopädische Fußchirurgie, München, Deutschland

***Question*** There is still a controversial discussion as to the treatment of talocalcaneal coalition with and without planovalgus deformity in young patients with only small numbers of treated patients in most of the published literature. Especially the percentage of unfavourable results seems to be a matter of major concern.

***Methodology*** From 2002 to 2014 80 cases (69 patients) of talocalcaneal coalition in children and adolescents under 18 years of age were treated with resection, tarsal osteotomy or primary fusion by a single surgeon. Treatment results were analyzed retrospectively with evaluation of the files completed by phone contacts in those who were lost to follow-up early. Thereby short to medium-term results (at least above 12, 36 months average) could be recorded of all but one patient. In 31 cases treatment consisted of resection and fat-grafting (group 1), in 26 additional cases tarsal osteotomy was added because of rigid planovalgus-deformity (group 2), and in 23 cases primary fusion of the talocalcaneal joint was performed.

***Result*** In group 1 one patient was lost to follow-up early with a severe peroneal spasticity and unknown further course. Two further patients still had remarkable pain after 15 months and another patient had to be fused 2.5 years after primary resection because of persisting pain. In group 2 secondary fusion was undertaken in two cases and offered in two other cases. Thus, among the patients with resection (group 1 and 2) there were 12.3 % unfavourable results in the short to medium term. In group 3 one patient with bilateral treatment and severe overweight still suffered from moderate tarsal pain after one and two years respectively although complete fusion occurred. Another patient had partial loss of sensation of the medial plantar nerve. All other patients in all groups were painfree or nearly painfree at the last visit with marked improvement in comparison to the preoperative situation.
***Conclusion*** Surgical treatment of talocalcaneal coalition in young patients is successful in most cases, but unfavourable results are not uncommon and may require secondary fusion. Differential indication between resection, additional deformity correction or primary fusion seems to be most important.

***Foot deformities: Lecture VKO-160***

#### *Treatment of Ponseti-Clubfoot-Relaps with an Abduction-Nightcast Versus TAT*

A. Helmers[1], S. Mittelhaus[2]

[1]EWK Spandau, Kinderorthopädie, Berlin, Deutschland, [2]EWK Spandau, Orthopädie/Unfallchirurgie/Kinderorthopädie, Berlin, Deutschland

***Question*** Is it possible to reduce the TAT in cases with a clubfoot-relapse after regularly Ponseti-treatment?
***Methodology*** We started to use the Ponseti-Method for clubfoot treatment in February 2005. Our collective of treated clubfeet has been monitored since 10 years. Regularly Ponseti-treatment until the age of 4 years. In cases with a relapse we put on the regularly abduction-brace for another 6 or 12 months and monitored the feet after 3 and 6 months again. Patients with a second relapse after 12 month bracing time we started to treat them with an individual nightcast-brace. This brace was individual used for 1, 2 or 3 years. We monitored all patients with the Pirani score and later with a combination of Pirani and Demeglio Score.
We treated the cavus relapse with a short scotch cast lower limb. After 4 or 6 months the eqiunus and adducts relapse was treated with an over knee scotch cast only used as a night brace.
***Result*** A relapses after Ponseti-treatment is combined with the compliance using the abduction brace 12–14 h per night. Relapses with a non compliance rank to 80 % and with a good compliance to 6 %. With an individual nightcast-brace is it possible to decrease the indication of TAT in clubfoot relapses under 50 %. The individual nightcast-brace, wearing only over night, can reduce the cavus, the equinus and the adductus part of the foot. The function of the foot was increasing and all patients represented a good compliance to use the brace.
***Conclusion*** All patients represented a good compliance to use the individual nightcast-brace. Decreasing the indication of TAT lower than 50 %. The cast can reduce effectively the cavus, the equinus and the adductus part of the relapsed foot. The costs of such a cast is not reproduced in the DRG-System.

### Paediatric Orthopaedic Pitfalls: ePoster

***Paediatric orthopaedic pitfalls: ePoster VKO-201***

#### *Aneurysmal Bone Cyst of the Patella*

S. Salminen

UK Köln, Orthopädie, Köln, Deutschland

***Question*** An aneurysmal bone cyst is a benign aggressive bone lesion first described by Jaffe and Lichtenstein in 1942. It is typically an expansile osteolytic lesion consisting of blood-filled spaces and channels that are divided by connective tissue septa, which may contain osteoid tissue and osteoclastlike giant cells. Less than 1 % of all primary bone tumours are aneurysmal bone cysts. There are few reported cases of an aneurysmal bone cyst of the patella, which comprises only 1 % of all aneurysmal bone cysts, and can be secondary in association with chondroblastoma or giant cell tumour.
A case of a patellar aneurysmatic bone cyst and its treatment at our clinic is presented.
***Methodology*** A 16-year old boy with quadriceps atrophy was complaining of anterior pain of his swollen left knee. The infection markers were negative. Radiographs of the left knee showed an multiseptated osteolytic lesion involving the whole patella. MRI revealed large trabecular cyst with fluid level in the patella. There was cortical thinning, but no perforation which was also arthroscopically verified. The patellar lesion was treated with an intralesional curettage, cancellous bone grafting, and additional filling with Cerament.
***Result*** The histology result was an aneurysmal bone cyst. The healing of the lesion was uncomplicated without further swelling of the knee. The quadriceps strength and knee ROM improved. The outpatient controls have not showed any recurrence of the patellar aneurysmal bone cyst yet.
***Conclusion*** Patellar tumours are uncommon, but almost 75 % of these are benign. Only 1 % of all aneurysmal bone cysts occur in the patella. Benign chondroblastoma and bony giant cell tumor must be considered in the differential diagnosis of aneurysmal bone cyst. MRI can display the internal architecture of the tumor with the presence of mineralization, expansion of the bone and changes in the cortex.
Intralesional curettage and bone grafting are the preferred treatment for benign patellar tumours without articular involvement. Recurrence rate is reported to be higher in children younger than 15 years. The treatment of the patellar aneurysmal bone cyst in our patient was successful with diminished swelling and pain of the knee as well as improved knee ROM and quadriceps strength.

***Paediatric orthopaedic pitfalls: ePoster VKO-167***

#### *Osteopetrosis: Case-Report of Patient with Genua Valga et Recurvata and Subtrocanteric Femur Fracture, Treatment Options and Their Complications*

H. Lengnick[1], H. Klima[2], E. Payne[2]

[1]Ostschweizer Kinderspital St.Gallen, Kinderorthopädie, St.Gallen, Schweiz, [2]Ostschweizer Kinderspital, Kinderorthopädie, Claudiusstrasse 6, 9006 St.Gallen, Kinderorthopädie, St.Gallen, Schweiz

***Question*** We present the case of a 9-year old boy suffering from osteopetrosis, who developed genua valga at the age of 4 years, later on genua nd bilateral subtrochanteric femur fracture. Radiological alterations of bones were: increased bone mass and bone density, streaky bone structure, reduced intramedullary space and enlarged metaphysis of long bones.
In osteopetrosis osteoclast function is diminished leading to reduced bone resorption.
***Methodology*** At the age of four, genua valga were treated by temporary hemiepiphyseodesis of the proximal, medial tibia. Valgus deformation was corrected, but the boy developed genua recurvata with pathologic posterior tibia slope +20° on the right and +15° on the left side (physiological mean value −10°) 1 year later.
***Result*** While correction of the deformation was satisfying, technical procedure was complicated with breaking of drills, K-wires and screws due to pathologic bone rigidity. Genua recurvata recurred 4 years later.

At the age of seven the patient developed right hip pain after trampoline jumping. X-ray showed a non-dislocated subtrocanteric femur fracture, which was initially treated conservatively due to former technical challenges. Pain persisted and dislocation became obvious radiologically. While the left leg was compensatory more stressed, he developed a subtrocanteric fracture on the left proximal femur as well. Therefore osteosynthesis of proximal femur was necessary on both sides. While the right side showed stable healing after 6 months, three proximal screws on the left side broke. Re-osteosynthesis was performed to stabilize bone-nonunion, but subsequently coxa vara developed and screws cut out proximally. Valgisation of the proximal femur with lateral wedge removal and plate osteosynthesis was done with difficulties of bone drilling for the screws—nevertheless bone healing was reached after 6 months.
***Conclusion*** Osteopetrosis belongs to the group of sclerosing bone dysplasia characterized by diminished osteoclast mediated skeletal resorption. We present a patient with recurrent deformities and fractures of the proximal femur, which are characteristic in osteopetrosis. Operative interventions showed remarkable intra- and postoperative complications, therefore precise pre-operative planning is necessary. Intraoperative frequent change of drill bits and periodic cooling while drilling help to prevent bone necrosis and subsequent complications like implant loosening and screw damage, failure, fracture and infection.
***Paediatric orthopaedic pitfalls: ePoster VKO-194***

#### *Pronation Deformity of Forearm Following Fracture both Bones of Forearm: Relavence of Pronator Teres in the Management of Fracture*

T. Wolf, C.U. Dussa, L. Döderlein

Orthopädische Kinderklinik, Kinderorthopaedie, Aschau i. Chiemgau, Deutschland

***Question*** We would like to describe a case of malunited fracture both bones of forearm which resulted in an extreme pronation deformity of forearm resulting in disability in day-to-day life. We would like to highlight the role of Pronator Teres (PT)in the treatment of such fractures.
***Methodology*** A 14-year-old male patient presented with the complaint of inability to supinate his forearm. He sustained following a fall in 2005 a fracture of both bones in the prox. 1/3rd of right forearm. He was treated in a trauma hospital with Prevot nails and cast. Following removal of the cast he sustained another fall and refractured his forearm at the same place which was treated conservatively with plaster. History of the patient revealed no chronic illness apart from an asymptomatic situs invertus.
On clinical examination showed an obese, 14 year old, height 176 cm, weight 93 kg. The right arm was pronated and the left in neutral position. The Pronation/Supination 90-40-0. The elbow and wrist flexion–extension movements were full and free. The relation of the ulnar styloid to the radius was disturbed; however the relative lengths of ulna and radius were normal. Fingers and shoulder were normal. Left forearm was normal.

Radiological examination showed no abnormality. No CT scan for torsional profile of forearm bones was performed.
***Result*** A rotational osteotomy of both radius and ulna were undertaken in a single sitting to restore the supination of forearm. The radius was supinated 35° and ulna 70°. The osteotomy was fixed with a plate. Intraoperative pronation-supination range of motion was 15°/0°/30°. Postoperatively the forearm was immobilised in plaster of Paris for 6 weeks. Radiographs at 6 weeks showed signs of bony union. Active and passive supination and pronation movements were started in physiotherapy at 6 weeks.
***Conclusion*** The deformity following fracture of both bones of forearm is dependent on the location of fracture and its relation to the insertion of Pronator teres (PT) muscle. A fracture proximal to the insertion of PT leads to a supination of proximal fragment due to unopposed action of the Supinator and Biceps muscle. While a fracture at or distal to the insertion of the PT, leads to a neutral position of the proximal fragment, while the distal fragment is pronated due to unopposed action of Pronator quadratus. This must be kept in mind during the conservative treatment or operative treatment of fracture of radius or both bones with intramedullary non-locking nails.

### Paediatric Orthopaedic Pitfalls: Lecture

***Paediatric orthopaedic pitfalls: Lecture VKO-132***

#### *A Minimum of 2 year Follow up of 22 EOS Patients Who Were Treated With 2nd Generation MCGR*

K. Ridderbusch[1], M. Rupprecht[1], P. Kunkel[2], C. Hagemann[2], R. Stücker[1]
[1]Altonaer Kinderkrankenhaus, Kinderorthopädie, Hamburg, Deutschland, [2]Altonaer Kinderkrankenhaus, Pädiatrische Neurochirurgie, Hamburg, Deutschland

***Question*** Growth-sparing techniques have changed our treatment of EOS significantly. Traditional Growing Rods require repeated surgical lengthenings under anaesthesia. This is the first single center report with more than 2 years follow up after treatment with magnetically controlled GR with a double rod technique.
***Methodology*** There were 22 EOS patients with a minimum follow up of 2 years (Ø 31, 24–46 months) and at least 6 (6–11) transcutaneous lengthening procedures. Only patients who with second generation rods were included. There were 7 patients with neuromuscular, 4 with NF, 6 with syndromes, 1 congenital, 2 thoracogenic and 2 idiopathic scoliosis. The average age of the 15 female and 7 male patients was 8.8 (4.6–14.3) years. Corrections of the primary curve and after lengthening were measured. We used the Cobb angle, and T1–T12 and T1–S1 as outcome parameters. Intra-and postoperative complications were recorded.
***Result*** The average curve measured 61.4° (40°–96°) and improved to 28.1° (11°–53°) after the index procedure representing a correction of 54 %. ($p < 0.05$) After at least 2 year follow up the average Cobb angle at most recent lengthening was 26.9° (11–64°). The average pre-operative T1–T12 length measured 183 mm (131–234) and increased to 204 mm (152–259) ($p < 0.05$) immediately postoperatively and 225 mm (174–282) ($p < 0.05$) at final lengthening. The average pre-operative T1–S1 length measured 296 mm (217–377) and increased to 333 mm (261–415) postoperatively ($p < 0.05$) and 355 mm (282–437) at final lengthening ($p < 0.05$). Overall 8 patients had at least one complication. This led to revision surgery in 7 patients. In two patients a pin within the actuator broke resulting in failure of the lengthening mechanism. Four patients developed a junctional kyphosis, two patients a screw pull out, one patient a lumbar adding on, and another one a deep wound infection.
***Conclusion*** Our 2 year FU results of MCGR in 22 patients with EOS have demonstrated that the MCGR is a safe and effective non fusion technique. MCGR provided adequate distraction similar to standard GR. The magnetically transcutaneous lengthening allows non-invasive distraction achieving comparable spinal growth to conventional GR. Complication were seen in one-third of the patients, however, compared to conventional GR the complication rate seems to be acceptable. Longer follow-up is needed to assess if these promising results can be maintained and especially if autofusion can be avoided.

***Paediatric orthopaedic pitfalls: Lecture VKO-163***

### *Analysis of Complications and Pitfalls of the LCP Pediatric Hip System*

M. Rogalski, M. Deja, K. Fliesenberg

Helios-Klinikum Berlin Buch, Kinderorthopädie, Zentrum für Orthopädie und Unfallchirurgie, Berlin, Deutschland

***Question*** Locking plate application is well established in adult orthopaedic trauma surgery. Reports for LCP-Systems in paediatric orthopaedics where published with different results. Especially in patients with reduced bone stability the angled blade plate requires safe retention and longer immobilisation periods to achieve healing without loss of correction or implant dislocation. The LCP Pediatric Hip System was developed for adequate reduction and protection of soft tissue. This system is adapted to the anatomy of children and adolescents as internal fixator of correction osteotomies of the proximal femur as well as stabilization of fractures. We ask for potential disadvantages of its use.
***Methodology*** Retrospective analysis of paediatric orthopaedic patients who underwent femoral osteotomy or fracture treatment between 08/2008 and 09/2014 and osteosynthesis with an LCP-Hip-System in a single center. We investigated operative procedure, bone healing, implant failure, loss of reduction and general complication rate and implant costs.
***Result*** 78 patients (59 CP, 3 MMC, 3 AMC, 3 NF I, 1 Larsen Syndrome, 2 Epiphysary Dysplasia, 5 Trisomy 21, 4 OIC) with a mean age of 7 years [4–19] and 96 correction osteotomies and 8 fracture osteosyntheses were included. 52 patients had unilateral, 26 patients single-stage bilateral procedures. The mean operational time was 54 min [46…120] for a single unilateral procedure and 118 min [96…165] for bilateral OT. The mean amount of intraoperative blood loss was 150 ml [90–280 ml] for unilateral procedures. All patients achieved radiologic fusion within 3 months. None of the hips showed a loss of reduction or implant failure. In 5 cases revision due to a fracture at the former osteotomy site following implant removal after 6 months was necessary. 1 case of fracture below the plate was recorded (OIC). 5 were CP-Patients with open wedge procedures. 7 patients developed hypertrophic scares, 2 haematoma but no other complications occurred.
***Conclusion*** With the LCP Pediatric Hip System one can achieve a stable reduction of the proximal femur. It allows an early postoperative mobilization in most of children. Caused by more rigid fixation, weak individual bone conditions and mode of operative procedure clinical stability after early hardware removal might be lesser despite radiological signs of consolidation. In these patients we can modify the operative procedure and recommend an implant removal not earlier than 1 year.

***Paediatric orthopaedic pitfalls: Lecture VKO-121***

### *Arthrodiatasis with Hinged Monoplanar External Fixator for Perthes Disease: A Technical Tip. Unpopular does not Mean Ineffective*

E. Cohen

Soroka Medical Center, Ben Gurion University, Orthopaedics, Beer Sheva, Israel

***Question*** To date, a consensus on the etiology and treatment of LCPD has not yet been reached. Hinged distraction of hip joint is an unpopular treatment option in Perthes Disease (LCPD) The procedure is called arthrodiatasis.
We used for hip arthrodiastasis the Orthofix(Pro Callus) fixator. We observed that application may be more exact by a minor change in technique. We postulate that an articulated device with less mechanical stress on the implant may prevent breaking the pins and less stress on the bone reduces pain. We propose a change in the technique of application of the fixator to improve the position of the hinge and add containment of femoral head.
***Methodology*** We changed the technique described in the implant instruction manual and elsewhere and placed the guide wire 5 mm distal to the femoral head center, while holding the femur in 15° abduction. Arthrodiastasis implies distraction, so in this manner at the end of the procedure, the femoral head will be displaced distally. Doing so the hinge and center of hip rotation will be colinear. Because the hinged fixator is finally applied in 15° hip abduction—(the maximum the actual design of Orthofix fixator allows)—the containment of femoral head is improved. Finally the Orthofix fixator is locked and distracted. If done correctly, at the end of the procedure with the patient still under anesthesia we will observe that is possible to achieve at least 80° of hip flexion without any stress.(Images 01,02)The proposed modification is applicable not only to Orthofix but to every type of hinged fixator.
***Result*** We used the technique in 6 cases in children 6–12 years old and diagnosed with LCPD. Obvious femoral head deformity was ruled out by MRI or arthography. The objective of the surgery was to prevent deformation of the femoral head by unloading the hip as long as possible. The fixator was kept for 4–6 months. The last follow up examination was done 3 to 8 years after surgery. The clinical and radiographic results were good. For clinical assesement patient acceptance and final Iowa hip score were counted. The radiographs were assessed by Stulberg classification and Epiphyseal Quotient.
***Conclusion*** The described modification in technique contributed to the promising results of arthrodiatasis. The procedure is minimally invasive and may prevent deformity of femoral head in selected cases of LCPD.

***Paediatric orthopaedic pitfalls: Lecture VKO-206***

### *Body Height Prediction and Detection of Growth Patterns after Systemic Chemotherapy in Children with Osteo- and Ewing's Sarcoma*

U. Lenze, K. Prautsch, A.H. Krieg

Universitäts-Kinderspital beider Basel, Basel, Schweiz

***Question*** The introduction of standardized chemotherapy protocols for the treatment of primary malignant bone tumors lead to a massive improvement in the survival of these patients. The effect of chemotherapy on growth development has not been uncovered until today. The multiplier method is a widespread method for height prediction in children but was shown to be not an appropriate tool for patients with Osteo- and Ewing's sarcoma at he time of initial diagnosis. The aim of this study was to analyse wether the multiplier method can be used at different time points of the treatment. Furthermore we aimed at evaluating if there are characteristic growth patterns after systemic chemotherapy
***Methodology*** In total 26 patients with Osteosarcoma or Ewing's sarcoma and an age at initial diagnosis below 14 years in girls and 16 years in boys were included. The multiplier method was used for prediction of the expected body height at maturity depending on the body height at various time points of the treatment (initial diagnosis, end of chemotherapy, 1/2/3 years after end of chemotherapy) and compared to the measured true body height at maturity. Additionally, depending on the body height at initial diagnosis the expected body heights at the above mentioned time points of the treatment were calculated and compared to the true body heights at these time points. Finally, all documented body heights were drawn into individual percentile curves and analysed with regard to possible growth patterns.
***Result*** All but one patient with Ewing's sarcoma achieved a lower body height then calculated at the time of initial diagnosis. In patients with Osteosarcoma, the body height at maturity was overestimated in 62.5 % and underestimate in 37.5 %. While the growth of patients with Ewing's sarcoma was parallel to the percentile at the time of initial diagnosis but stopped with an early age, there was a growth arrest in patients with Osteosarcoma until 1 year after end of chemotherapy followed by a catch up phase.
***Conclusion*** Our results suggest, that there are characteristic growth patterns following chemotherapy especially in patients with Ewing's sarcoma. The multiplier method is not a reliable growth prediction tool at the time of initial diagnosis but—at least in patients with Ewing's sarcoma—seems to be appropriate 1 year after end of chemotherapy. Further studies with higher numbers of patients are needed to confirm these results and calculate corrections factors for the conventional multiplier method.

***Paediatric orthopaedic pitfalls: Lecture VKO-180***

### *Diastrophic Dysplasia: a Long Term Follow-up of Orthopedic Pediatric Treatment in 26 Cases*

J.K. Correll, M. Salzmann

Klinikum Rechts der Isar, TU München, Kinderorthopädie, München-Schwabing, Deutschland

***Question*** Diastrophic dysplasia belongs to the large, heterogenous group of skeletal dysplasias.
Patients present at birth with short stature, disproportion, complex deformities of the lower limb, the characteristic hitch hiker thumb and rigid diastrophic clubfeet. Caused by a defect in a sulfate transporter gene, the structure of cartilage is abnormal and leads to early degeneration. All patients develop increasing contractures, especially of the weight bearing lower extremities. Regardless whether they were operated on, the deformities are progressive. Most patients loose their ability to walk even in young age. There are only few reports in literature and no recommended treatment up to now.
***Methodology*** In cooperation with the BKMF (Bund kleinwüchsiger Menschen und ihrer Familien) we are able to report the results of orthopedic treatment in 26 patients with the Caucasian diastrophic dysplasia mutation. Patients with the milder Finnish mutation were excluded. Clinical and radiological evaluation of the orthopedic problems as foot deformities, development of contractures, early degeneration and deformity of the joints, ability to walk, results of operative and conservative treatment and incidence of complications were documented, also the patients' subjective contentment. Photo documentation was done pre- and postoperatively. The mean follow up was 15.3 years, in two patients we can report the treatment results of more than 30 years.
***Result*** Foot deformities in these patients can't be compared with normal clubfeet. Operative treatment must be more aggressive caused by bony deformations. The Ponseti method is not adequate. All patients developed severe flexion contractures especially of the hip and knee joints. At adolescence there was no difference between conservative treatment and surgery in all patients. However increase of flexion contractures and the deformation of hip and knee joint were notably correlated with body weight. Two patients were painless and could walk about 1 h 5 years after bilateral total hip replacement.
***Conclusion*** Operative treatment of flexion contractures of the lower limb should be considered strictly. There is no evidence for better results in the history of the disease.
Foot deformities can be corrected surgically but they need an aggressive approach and a careful aftercare.

***Paediatric orthopaedic pitfalls: Lecture VKO-202***

### *Paediatric Orthopaedic Evaluation of Children Suffering from Nephropathic Cystinosis at the Interdisciplinary Cystinosis Clinic in Traunstein*

N. Herzig[1], U. Treikauskas[2], A. Rausch[3], G. Steidle[4], R. Brangenberg[2], H. Hintner[5], R. Aghayan-Ugurluoglu[6], U. Römer[2], L. Treguer[7], M. Haselsberger[7], M. Passow[2], O. Greil[8], C. Köppl[4], I. Landthaler[9], C. Knerr[10], E. Harms[11], P. Bernius[1], K. Hohenfellner[2]

[1]Schön Klinik München Harlaching, Kinder- und Neuroorthopedics, München, Deutschland, [2]Klinikum Traunstein, Klinik für Kinder- und Jugendmedizin, Traunstein, Deutschland, [3]Praxis, Kinder- und Jugendmedizin, Traunstein, Deutschland, [4]SPZ Traunstein, Traunstein, Deutschland, [5]Paracelsus-Universität-Salzburg, Dermatologie, Salzburg, Österreich, [6]Paracelsus-Universität-Salzburg, Augenheilkunde, Salzburg, Österreich, [7]Klinikum Traunstein, Nephrologie, Traunstein, Deutschland, [8]Klinikum Traunstein, Radiologie, Traunstein, Deutschland, [9]Ernährungsberatung, München, Deutschland, [10]Logopädie, Traunstein, Deutschland, [11]Universitätsklinikum, Münster, Deutschland

***Question*** Cystinosis is a very rare lysosomal storage disease, which follows an autosomal recessive inheritance pattern. Prevalence in Germany is 1:100.000–200.000. The most frequent type is infantile nephropathic cystinosis, a multiorgan disease, in which cystine is stored in most organs. Losses of function arise with increasing age and concern the kidneys and eyes followed by the musculoskeletal system, endocrinal organs and CNS. In 2012 an interdisciplinary cystinosis clinic was established in Traunstein in order to consider all aspects of this systemic disease for all age groups. Patients go through the following specialist depts in 2–3 h: Nephrology, orthopaedics, physical therapy, cardiology, pulmonology, gastroenterology, neurology, ophthalmology, endocrinology, dermatology, nutrition consultation, speech therapy. Between 10/2012 and 10/2015 149 patient contacts (62 individual patients) took place. The orthopaedic findings of 33 paediatric patients are presented in the following paragraphs.
***Methodology*** The results were evaluated at the time of first contact. 15 boys and 18 girls (aged 14 months–17 years) were included. The time of initial diagnosis with these children lay between 5th day of birth and 8 years (average age 2.4 years).
***Result*** Only 2/33 children were without orthopaedic findings (age 22 and 36 months). 31/33 presented various deformities: 21 pes valgus, 16 leg-deformities, 14 genua valga and 2 genua vara with rickets. 13 sagittal spinal deformities and 5 scoliosis, 4/5 requiring a corset, 2 already supplied. 7/33 children complained of regular pain. 4/33 have undergone surgery for correction of flatfeet and/or leg axis. 4/33 children suffered from rickets. In older teenagers muscular atrophies occurred as indication of muscle wasting.
***Conclusion*** The introduction of the interdisciplinary cystinosis clinic with a fixed team of specialists proved to be successful for this rare systemic disease. Due to the high number of patient contacts within a short period of time, important clinical experience can be collected. From the perspective of orthopaedics, the focus was on deformities and pain. Therefore regular evaluation of the musculoskeletal development of children suffering from cystinosis is of great importance in order to identify those factors that have an influence on the development of orthopaedic deformities. The structured and continuous collection of data from all of the above mentioned specialist depts will contribute to a better quality of treatment for these patients.

***Paediatric orthopaedic pitfalls: Lecture VKO-149***

### *Surface Optometry in Scoliosis Comparison of 102 Scoliosis Patients and a Peer Group of 100*

E. Dingeldey, J. Willers, I. Barsikow, F. Wagner, J. Matussek

Universität Regensburg, Orthopädie, Bad Abbach, Deutschland

***Question*** Surface optometry is used as a control parameter in observation of scoliosis.
The aim of this study was to find out whether back shape analysis with surface optometry is an appropriate method to detect scoliosis in children and teenagers. The second goal was to determine how symmetrical the backs of the general population are. Therefor we focused on surface rotation, pelvic obliquity and plumb line. In the questionnaire we concentrated on previous treatment and sport activities.
***Methodology*** This study includes 102 patients with scoliosis and a peer group of 100 children and teenagers without any known spine deformities. Both groups were given a questionnaire, were clinically examined and surface optometry was performed. In addition X-rays of all 102 included scoliosis patients of our outpatients section were available.
***Result*** The scoliosis group showed a wide range of surface rotation, which was not really correlated with their Cobb angle of scoliosis but with the treatment, especially bracing and physiotherapy, they already got. Patients with good correction potential achieved as good back shape parameters as the average of the peer group.
The peer group as well showed a wide range of surface rotation. Although we only discovered scoliosis and asymmetries in some patients in clinical examination, all showed at least some rotation of their back and none of them was completely symmetric.
***Conclusion*** As a conclusion we would not appreciate to use rasterstereographic back shape parameters as a screening method by itself but as an additional verification.
Furthermore we found out that the average population is not as symmetric as we thought but has a slight asymmetry.

***Paediatric orthopaedic pitfalls: Lecture VKO-107***

### *Ultrasound Diagnosis of Fractures: State of the Art*

O. PD Dr. med. Ackermann[1], K. Eckert[2]

[1]EVK Mettmann, Mettmann, Deutschland, [2]Elisabeth-Krankenhaus, Essen, Deutschland

***Question*** For the reason that the growing patient is ten times more vulnerable to ionizing damages than adults, it is a universal aim to minimize the usage of radiation in diagnostics. Recent research showed that ultrasound may be a viable alternative to plain X-rays in certain indications. The aim of the lecture is to define safe and applicable indications for ultrasound imaging in order to reduce the X-ray exposition of children and adoslecents.
***Methodology*** A review of the recent literature and the personal experience of the authors is the basis for the definition of certain fields of application of ultrasound in fracture diagnosis. The technique is explained, opportunities and hazards are defined as well as the requirements to ultrasound device and examiner. The safeness and feasibility must be similar to X-ray diagnosis.

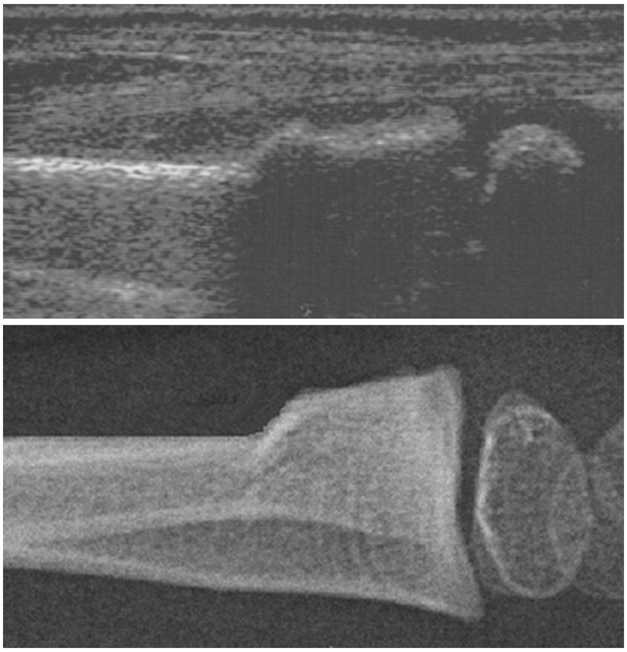

***Result*** Three application fields are defined:
(a) the forarme fracture can be easily visualized by ultrasound means, an X-ray is not necessary unless an operation is planned. Radiation exposure can be reduced by 90 %.
(b) elbow fracture can be ruled out by diagnostics of the dorsal fat pad sign. In any case of doubt or a positive fat pad sign, X-ray is recommended. This leads to a reduction of X-rays of 70 %.
(c) proximal humerus fractures can be ruled out by ultrasound; in case of a fracture the deformation is more precisely determined by US in contrast to X-rays in two planes.
The examination technique is easily applicable for every surgeon, even with no specialization in ultrasound. A standard ultrasound device with a linear transducer is applicable.
***Conclusion*** In the most common indications of traumatology in children (forearm and elbow) ultrasound allows a significant reduction of radiation exposure. In proximal humerus fractures the quality of diagnosis is better than with X-ray Imaging. The method is applicable even for surgeons without specialization in ultrasound imaging.

## How-to-treat Cases: Lecture

***How-to-treat cases: Lecture VKO-119***

### *Neglected Hip Fracture after Percutaneous Fixation of Epiphysiolysis Cap. Fem. in a 10 years Old Girl*

H. Gaulrapp

FAcharztpraxis für Orthopädie und Kinder-Orthopädie, München, Deutschland

***Question*** A 10 year old girl had received percutaneous fixation of a mildly slipped femoral epiphysis of her left hip joint together with a preventive fixation of her uninjured right hip. 4 months later she presents herself after having fallen and injured her right hip 2 weeks earlier. She can put weight on her injured leg but shows slight pain on moving her hip joint. X-ray reveals a lateral non-displaced fracture.

***Methodology*** As she denied immediate operation, non-weightbearing was recommended and an e-mail-based survey conducted, enrolling 11 national specialists in pediatric orthopedics and traumatology to obtain an expert basis for treatment.
***Result*** Results after 2 days showed extremely contradictory recommendations (second screw, plate, casting, continuation of non-weightbearing) which led to going on "doing nothing" and a very pleaeant final result.
***Conclusion*** This case is to be presented as an outpatient "how to treat?" case using e-mail-consultation for saving time and ways for the patient and distributing a variety of helpful but contradictory treatment advices, helping the patient to select his individual treatment.

***How-to-treat cases: Lecture VKO-195***

### *Nora's Lesion on the Tibia of a 4 Years Old Boy*

C. Polan, D. Spodeck, M. Hövel

Universitätsklinikum Essen, Klinik für Orthopädie und Unfallchirurgie, Essen, Deutschland

***Question*** Our objective is to present the Norás lesion, which is a bizarre parosteal osteochondromatous proliferation.
***Methodology*** In February 2013 the 4 years aged Antonio was presented in our orthopedic policlinic. Half a year ago the mother had noticed a knot at the left lower leg. The boy didn̈t feel pain, he had no antecedent trauma.
Clinically we found a hard tumor at the distal tibia. The surrounding soft tissue was not altered. He had a normal gait without limping.
X-ray and MRI-pictures showed a $3 \times 2 \times 1.5$ cm$^3$ calcified tumor sitting dorsomedial on the diaphysis of the left tibia.
Isotope bone scan showed increased uptake beside the diaphysis of the tibia [4].
The biopsy offered an unusually big bizarre parosteal osteochondromatous proliferation (Nora's lesion).
In April 2013 we removed the whole tumor. The histology was confirmed. 2 1/2 years later, in December 2015 there is no sign of recurrence.
***Result*** Bizarre parosteomal osteochondromatous proliferation described by Nora and colleagues in 1983 (also called Norás lesion) is a very rare benign tumor [1, 2]. It is an exophytic outgrowth from the cortical surface consisting of bone, cartilage and fibrous tissue.
It usually affects the phalanges, and the metacarpal or metatarsal bones [3]. It affects patients of any age, but more in their 20 and 30 s. It has a very high recurrence rate.
***Conclusion*** A Nora's lesion on the tibia of a young child is not yet published.

***References***
[1] Nora FE, Dahlin DC, Beabout JW, (1983), Bizarre parosteal osteochondromatous proliferations of the hands and feet., Am J Surg Pathol. 1983 Apr;7(3):245–50
[2] Meneses MF, Unni KK, Swee RG, (1993), Bizarre parosteal osteochondromatous proliferation of bone (Nora's lesion)., Am J Surg Pathol. 1993 Jul;17(7):691–7
[3] Mahajan S, Chandra R, Mohan Lal Y, (2012), "Nora lesion" - Bizarre parosteal osteochondromatous proliferation., J Clin Orthop Trauma. 2012 Dec;3(2):119–21
[4] García-Alvarez F, Lacleriga AF, Bueno AL, Castiella T, Seral F, (1999), Bizarre parosteal osteochondromatous proliferation. Difficulty in diagnosis., Chir Organi Mov. 1999 Apr-Jun;84(2): 179–82

***How-to-treat cases: Lecture VKO-189***

### *Osteofibrous Dysplasia Campanacci of the Ulna*

M. Hövel, C. Polan

Universitätsklinikum Essen, Klinik für Orthopädie und Unfallchirurgie, Essen, Deutschland

***Question*** Our objective is to show that the osteofibrous dysplasia Campanacci can involve the ulna.
***Methodology*** Osteofibrous dysplasia was described as a variant of fibrous dysplasia by Campanacci in 1976 [1]. The tibia is almost always involved, the fibula may exceptionally be affected. The osteofibrous displasia becomes symptomatically and is usually diagnosed in the first decade of life.
The characteristic radiographic finding is an eccentric intracortical osteolysis with varying expansion in the diaphysis of the tibia.
Histologically the irregular spicules of trabecular bone are lined with osteoblasts [2].
***Result*** Hamza lived in Aleppo in northern Syria. In 2010, aged 5 years, Syrian surgeons filled a cystic lesion in the right ulna with unknown substitute material.
Now, 10 years old, we started our orthopedic treatment, because the pain in his forearm increased again.
Clinically the ulna offered a prominent aching bowing.
The biopsy showed the characteristic pattern of an osteofibrous dysplasia. We resected the extended lesion and filled it with cancellous bone from the ipsilateral pelvic rim.
***Conclusion*** Looking intensively at the literature, we found that only four publications dealt with osteofibrous dysplasia of the ulna. Only one case was described in the radius.

***References***
[1] Campanacci, M., (1976), Osteofibrous dysplasia of long bones. A new clinical entity., Ital. J. Orthop. Traumatol., 2:221,1976
[2] Tachdjian, Mihran O., (1990), Pediatric orthopedics, W.B. Saunders

## How-to-Treat Cases: How to Treat: Case Description

***How-to-treat cases: How to treat*****:** ***Case description VKO-135***

### *Case Report: Infant from Libya with Amputation of the Lower Leg and Pseudarthrose of the Femoral Bone after Gunshot Wound*

V. Frimberger, M. Poschmann, P. Bernius

Schön Klinik München Harlaching, Zentrum für Kinder- und Neuroorthopedics, München, Deutschland

***Question*** Gunshot wounds in infants are a rare entity in Germany.The difficulty treating such injuries is due to extensive damages of the soft tissue with consecutive dysfunction of perfusion and innervation, osteal defects and deep contamination with colonization. The interdisciplinary teamwork of all involved departments is essential to provide a sufficient rehabilitation.
***Case Presentation*** We report on a boy with the age of 2 years and 8 months who suffered from a full penetration gunshot of the left femoral bone in Libya in Sep. 2011. The initial medical care was provided locally. After being transferred to Tunisia for further care, one had to ablate the lower leg 8 cm below the left knee as a result of circulatory disturbance. For stabilisation a external fixateur was installed. With the help of an aid organisation the boy was brought to Germany in October 2011.
After unpacking the leg the diagnostic findings presented a badly groomed fixateur which bridged the osteal gap of the distal femur. The skin of the stump was roughly adapted by a few stitches below the knee. The soft tissue was suchlike destroyed that one could look right through the thigh showing the osteal stumps.
Primarily the treatment focused on the soft tissue damage, which is a necessary precondition before a osteal consolidation can happen. So the external fixateur was removed, the bone stumps shortened and a vacuum dressing applied. Over a period of 4 weeks and after nine operations, including a skin graft, wound and stump closure was achieved. The child then was transferred to a paediatric orthopaedic department, where the treatment of the femoral fracture and a residual and discharging fistula was approached. For the osteosynthesis of the femoral fracture two ESINs were used. Despite the radiological findings of a pseudarthrose, soon an osteal consolidation process emerged, caused by the enormous stimulus of the healing soft tissue defect. Finally we manufactured a transtibial prosthesis in our orthopaedic workshop, so the boy could be mobilised.
***Conclusion*** Devastating injuries of the limbs caused by gunshots in infants and children are a rare entity. Their treatment represents a major challenge for all involved therapists. From our medical point of view it is indispensable, at first to reconstruct the soft tissue and secondly to handle the osteal complex of problems. This requires high standards and a comprehensive concept from both physicians, qualified nurses, physiotherapy and orthopaedic engineering.

***How-to-treat cases: How to treat*: *Case description VKO-205***

### *Complex Reconstruction of the Hip Joint after Purulent Coxitis with Coincidence of DDH with Coxa Magna in the Contralateral Hip*

H. Tretow[1], R. Rödl[1], G. Gosheger[2], F. Schiedel[1], B. Vogt[1]

[1]Kinderorthopädie, Deformitätenrekonstruktion und Fußchirurgie, Universitätsklinikum, Münster, Deutschland, [2]Klinik für Allgemeine Orthopädie und Tumororthopädie, Universitätsklinikum, Münster, Deutschland

***Question*** We present the case of a little girl suffering from a complex deformity of the right hip joint due to a purulent coxitis caused by a newborn septicemia.
The destruction of the femoral head and dysplasia of the acetabulum resulted in a luxation of the hip at the age of 5 years. The femoral neck was in severe varus position, the head showed a grotesque shape.

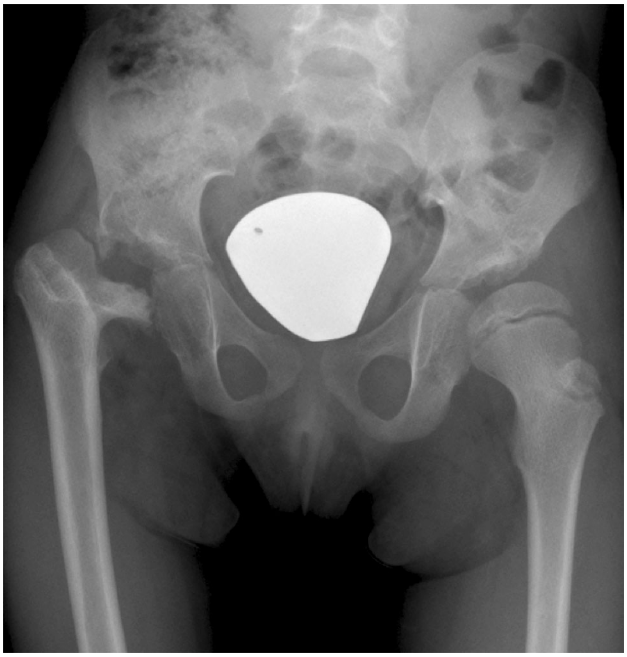

Reconstruction and reduction was achieved by valgisation osteotomy of the femoral neck, reshaping of the head and a shelf acetabuloplasty. While the right hip joint first shows good recovery, the left hip joint worsenes in terms of acetabular index and lateral migration as well as developing a coxa magna-shape.
After a varisation osteotomy on the left side, the right hip again causes problems due to a high-standing trochanter.
Also, the left hip shows a relapse of dysplastic acetabulum and increasement of lateral migration.
The girl now is 12 years of, suffering from a reduced ability of walking and positive Trendelenburg gait at the right.
At this point, we want to discuss future options for improving the situation and possibly preventing coxarthrosis.

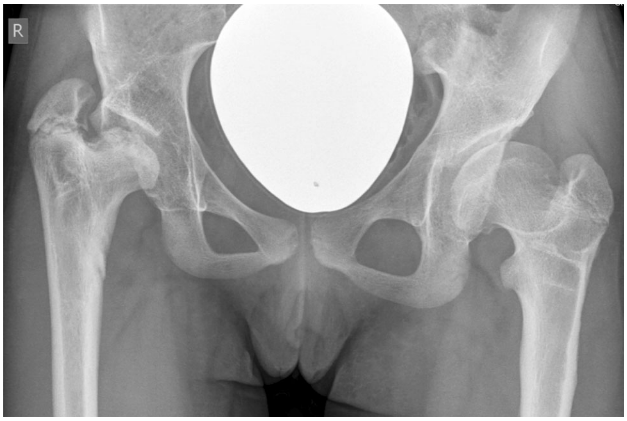

***How-to-treat cases: How to treat*: *Case description VKO-157***

### *Supramalleolar Valgus Deformity with Partial Necrosis of the Distal Tibial Epiphysis*

M. Nell, J. Hamel

Zentrum für Fuß- und Sprunggelenkschirurgie, Orthopädisches Zentrum Arabellapark, München, Deutschland

***Question*** Three cases of supramalleolar epiphyseal growth-disturbance with spontaneous partial necrosis of the distal tibial epiphysis with fragmentation in older children are presented.

In two cases the fibula showed slight shortening, in one case a slight external-rotation-disturbance of the tibia was found additionally. Two of three male children were previously healthy, the third one was suspected to suffer from a mild form of epiphyseal dysplasia because of additional varus-growth-disturbance around both knee joints.

Patients were treated with supramalleolar osteotomy and/or hemiepiphysiodesis.

Ankle valgus has been described in posttraumatic cases or neurological and bony disorders, but not in otherwise healthy children.

The etiology of ankle valgus, the role of fibular shortening and therapeutic aspects should be discussed, especially options of guided growth.

Open Access

This article is distributed under the terms of the Creative Commons Attribution 4.0 International License (http:// creativecommons.org/licenses/by/4.0/), which permits unrestricted use, distribution, and reproduction in any medium, provided you give appropriate credit to the original author(s) and the source, provide a link to the Creative Commons license, and indicate if changes were made.

